# Recent Advances of Atomic/Molecular Layer Deposition Engineering Silicon Interface for Lithium-Ion Batteries

**DOI:** 10.1007/s40820-026-02259-9

**Published:** 2026-06-22

**Authors:** Haocheng Wen, Yuhui Xu, Xiaoxue Wang, Ming Li, Lulu Zhang, Huaming Qian, Jia Kang, Xuexia Song, Yetong Li, Jingjing Wang, Jiujun Zhang, Xifei Li

**Affiliations:** 1 Institute of Advanced Electrochemical Energy and School of Materials Science and Engineering, Xi’an University of Technology, Xi’an, Shaanxi 710048 People’s Republic of China; 2 Shaanxi Engineering Research Center of Key Materials for Lithium/Sodium-Ion Batteries, Xi’an University of Technology, Xi’an, Shaanxi 710048 People’s Republic of China; 3 Institute of New Energy Materials and Engineering, College of Materials Science and Engineering, Fujian Engineering Research Center of High Energy Batteries and New Energy Equipment & Systems, Fuzhou University, Fuzhou, Fujian 350108 People’s Republic of China

**Keywords:** Atomic layer deposition, Molecular layer deposition, Si anodes, Lithium-ion batteries, Interfacial Engineering

## Abstract

Despite the ultra-high theoretical capacity of silicon (Si) anodes, their practical application is severely hindered by drastic volume effect and an unstable solid-electrolyte interphase (SEI). Atomic/molecular layer deposition (ALD/MLD) technology exhibits unique advantages in mitigating these challenges by constructing a stable and functionalized interface on Si anodes. This review summarizes recent advances and presents perspectives on ALD/MLD for Si anode interface engineering. Firstly, the existing interfacial challenges are analyzed: mechanical failure and structural degradation, complex/unstable SEI, and insufficient interfacial charge transfer. Subsequently, the key role and mechanism of ALD/MLD in enhancing the performance of Si anodes are elucidated from an atomic/molecular-scale interface engineering perspective. Crucially, three key aspects are emphasized: enhancing structural stability through mechanical confinement and stress dissipation, inducing the formation of stable SEI via ingenious interface chemical design, and constructing efficient ion/electron transport channels to optimize interfacial charge transfer kinetics. Finally, future research directions on ALD/MLD for Si anode interface engineering are proposed.

## Hightlights

- Reveals the chemical-mechanical coupled process of interfacial failure in Si anodes.
- Elucidates the core role of atomic/molecular layer deposition via atomic/molecular-scale interface engineering.
- Demonstrates how tailored interfaces solve stability, solid-electrolyte interphase, and kinetics problems.

## Graphical abstract

**Figure:**
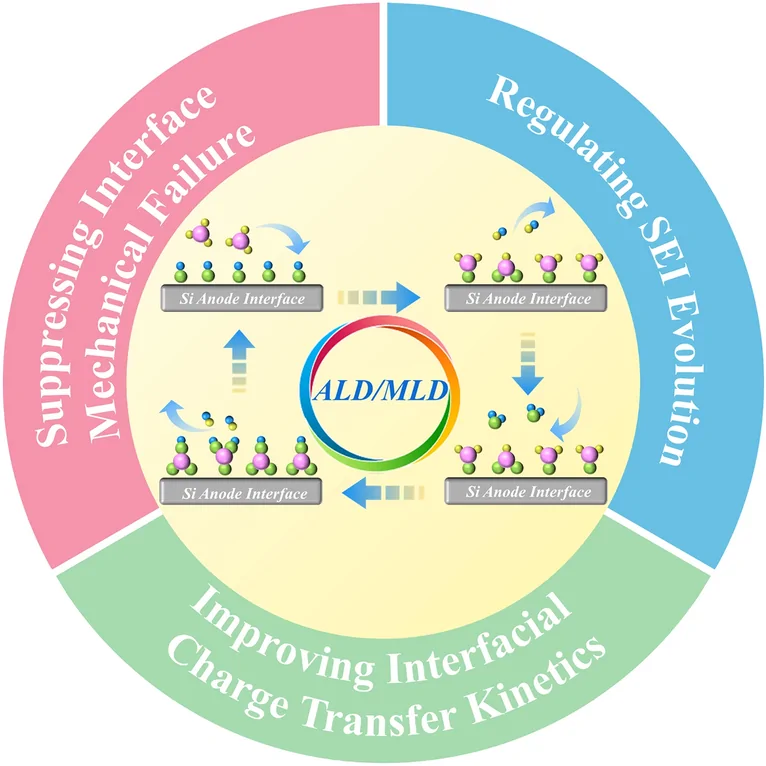

## Introduction

Lithium-ion batteries (LIBs) have achieved great success as energy storage devices. However, the conventional graphite anode can no longer satisfy the requirements of practical applications. Anode materials with a high capacity and desirable cycle stability are urgently needed for next-generation lithium batteries [1–4]. Si anodes are viewed as promising candidates, due to its excellent properties such as ultra-high theoretical specific capacity (3579 mAh g^−1^) and suitable operating potential (< 0.5 V vs. Li/Li⁺) [5–7]. However, the industrialization process of Si anode materials still faces many challenges: dramatic volume expansion (300% ~ 400%), low intrinsic electronic conductivity (10^−5^ ~ 10^−3^ S cm^−1^), low lithium-ion diffusion coefficient (10^−14^ ~ 10^−13^ cm^2^ S^−1^), and interface instability [8–13]. The first three issues can be partially addressed through ingenious structural design and the development of novel binder/electrolyte additives [14–18]. However, the problem of interfacial instability is particularly complex, stemming primarily from the significant volume changes of Si, which cause particle fragmentation, conductive network failure, and most critically, the continuous breakdown and regeneration of the solid-electrolyte interphase (SEI) film. This vicious “fracture-regeneration” cycle irreversibly consumes active lithium and electrolyte, accompanied by severe electrode polarization. It is the root cause of capacity degradation and poor cycle life [19, 20].

Over the past few decades, researchers have explored various coating techniques for the interface engineering of Si anodes [21–23]. Chemical vapor deposition (CVD) enables the formation of dense, high-purity coatings on planar substrates with advantages such as high deposition rates. However, it typically requires elevated temperatures (> 500 °C) and complex gas control systems, and exhibits limited conformality on complex three-dimensional structures [24]. Physical vapor deposition (PVD), including sputtering, produces films with high purity and density. However, as a line-of-sight technique, it inherently suffers from shadowing effects on non-planar substrates, leading to discontinuous coverage and often weak interfacial adhesion between the coating and the substrate [25, 26]. Wet chemical methods offer the advantages of simple processing and low cost, but their conformality on high-aspect-ratio structures is constrained by differences in solution reaction kinetics, resulting in uneven coverage and particle agglomeration due to weak interfacial adhesion [27, 28]. Consequently, these methods still face significant challenges in achieving conformal coatings on complex nanostructures, precise interface design, and compatibility with the requirements of durable Si anodes.

Atomic/molecular layer deposition (ALD/MLD) technology offers unique capabilities for the precise design and rational regulation of Si anode interfaces, owing to its atomic-level precision in film thickness control, unparalleled conformality, and flexible/adjustable chemical composition [29–33]. Crucially, the Si surface is covered by a native oxide layer (SiO_x_), which renders the surface rich in hydroxyl (−OH) groups. These groups provide ideal reactive sites for the self-limiting surface chemistry of ALD/MLD. During deposition, precursor molecules chemisorb specifically onto these surface −OH groups, thereby enabling uniform nucleation and layer-by-layer growth of the coating. Leveraging this inherent chemical compatibility, ALD/MLD technology enables the construction of various functional interfaces on complex three-dimensional Si surfaces, including rigid inorganic coatings, flexible organic–inorganic hybrid coatings, and even gradient composite structures that combine rigidity and flexibility. By precisely modulating the surface chemistry and interfacial architecture at the atomic/molecular level, ALD/MLD transforms the inherently unstable and dynamically evolving Si/electrolyte interface into a rationally designed, multifunctional interphase. This interphase synergistically accommodates mechanical strain, regulates SEI composition, and facilitates charge transfer, thereby providing a pioneering solution for high-performance Si anodes.

**Fig. 1:**
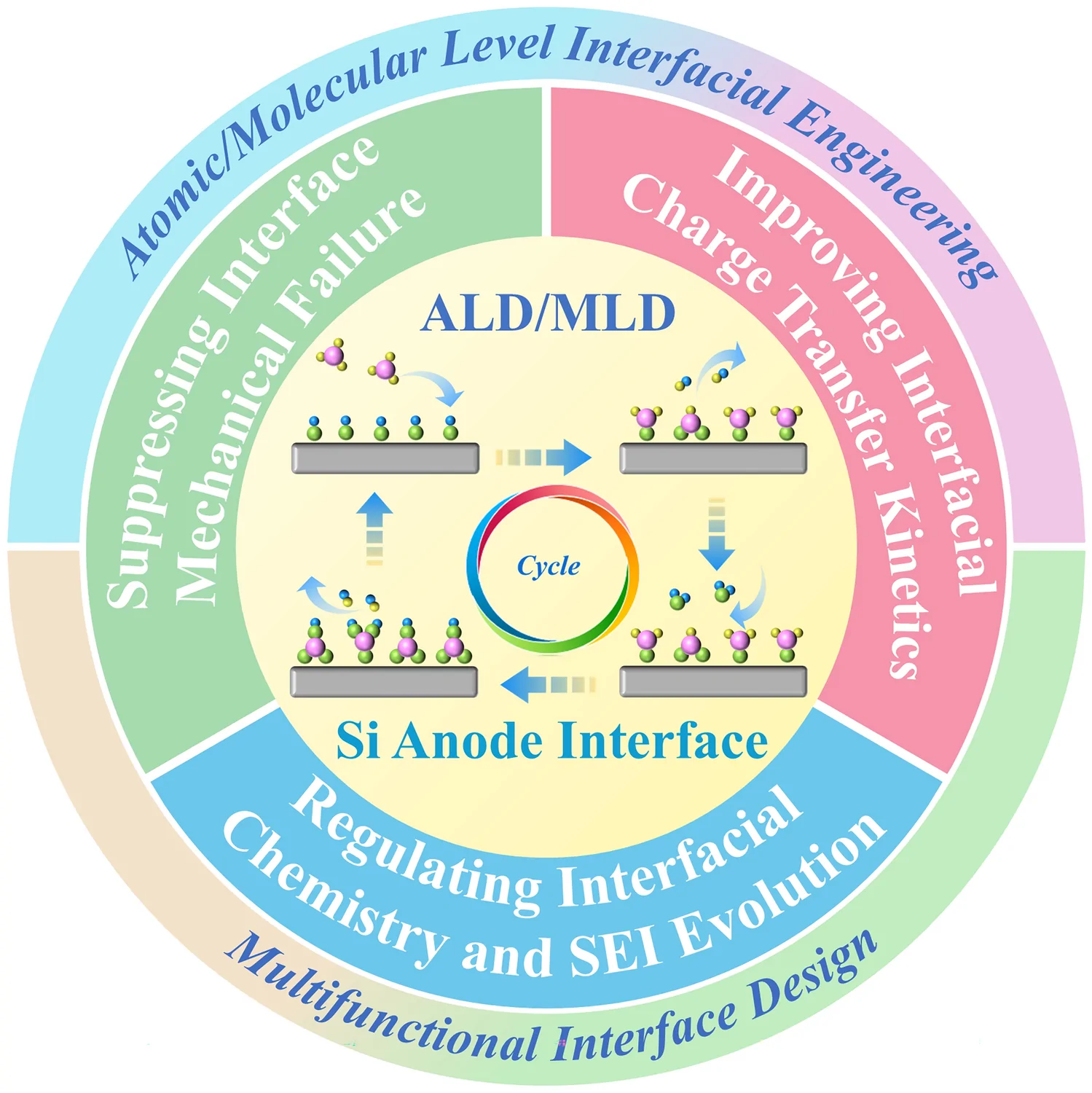
Overview of the core role and mechanism of ALD/MLD stable Si anode interface engineering

This review systematically summarizes the latest research progress of ALD/MLD technology in addressing the interface challenges of Si anodes. First of all, the intrinsic mechanism and core challenges of Si anodes interface failure are analyzed. Subsequently, it focuses on how ALD/MLD technology can suppress mechanical failure, regulate the evolution of SEI and improve the charge transfer kinetics through multifunctional interface design (Fig. 1). Finally, the core contributions of ALD/MLD technology are summarized. Its future development direction is projected, aiming to provide references for the development of high-performance and long-life Si-based anodes.

## Challenges of Silicon Interface

### Interfacial Failure Mechanisms of Si Anodes

**Fig. 2:**
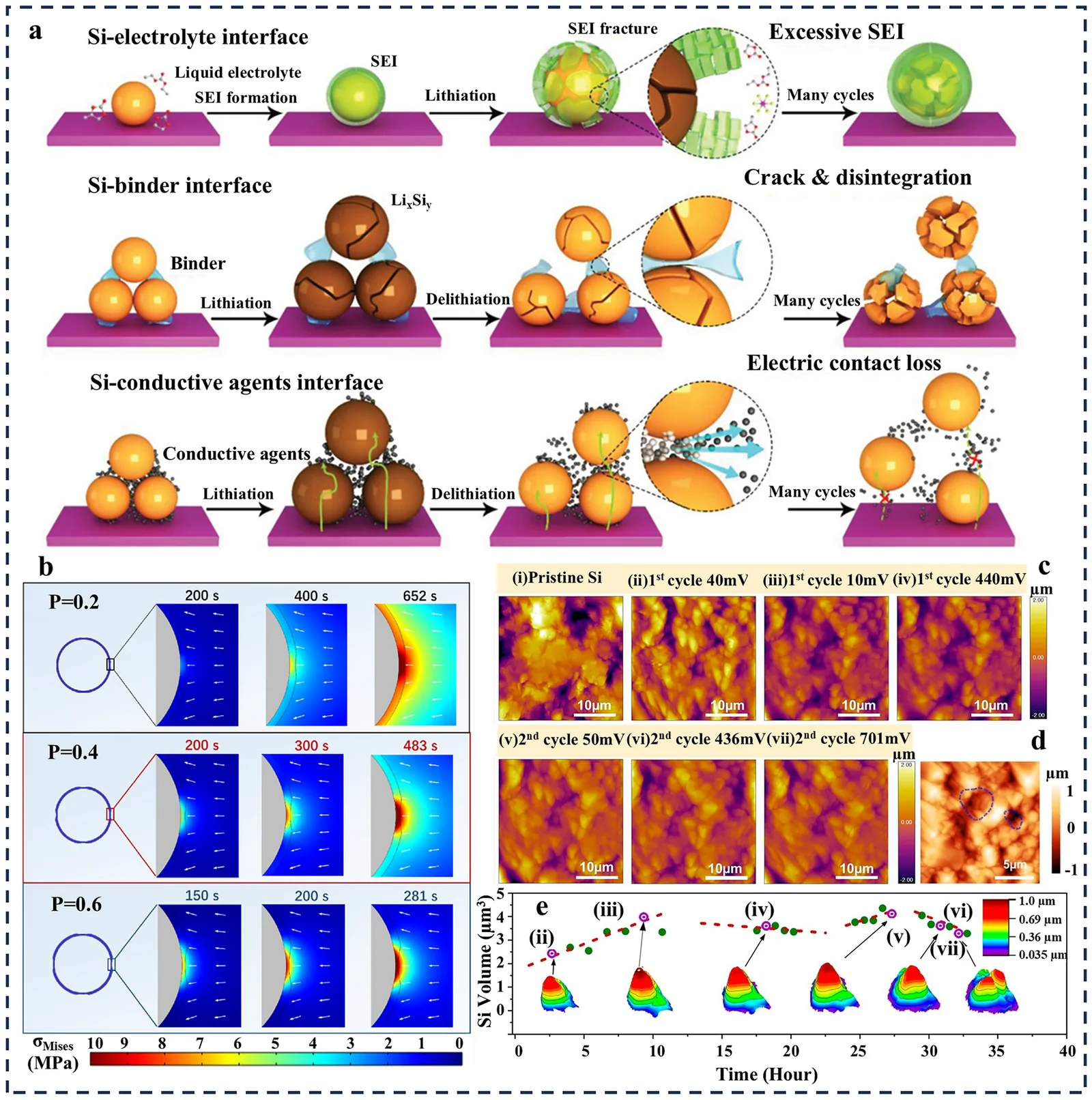
**a** Schematic representation of the interface failure mechanism of Si anodes. Reproduced with permission [37]. Copyright 2022, John Wiley and Sons. **b** Von Mises stress distribution in the right region of the SEI until structural failure. Reproduced with permission [43]. Copyright 2024, KeAi Communications Co. **c** In situ AFM images of μSi particles during the first two lithiation and delithiation processes. **d** AFM image of particle loss during Si electrode cycling. **e** 3D image of μSi particles and its volume change at each measurement point. Reproduced with permission [44]. Copyright, 2022 American Chemical Society

The main challenges of the Si anode during cycling can be attributed to the uncontrolled interface instability, volume effects, and the dynamic evolution of multi-interface synergy [34–36]. In recent years, researchers gradually reveal the interfacial failure mechanism of Si anode materials by adopting advanced characterization techniques. As shown in Fig. 2a, Si undergoes a structural transition from crystalline Si to amorphous Li_x_Si and Si during the lithiation/delithiation process. Anisotropic volume expansion and contraction lead to cracks and pulverization within the particles, and this volume effect further induces multiple interfacial damage [37]. Firstly, the thin SEI layer ruptures and dissolves during delithiation shrinkage, exposing fresh Si surfaces and triggering persistent chemical side reactions with the electrolyte. This not only irreversibly consumes active lithium, but also causes the formation of a thick and loose SEI layer. Its composition changes from mainly organic components to a multi-layer heterogeneous structure rich in inorganic lithium salts (such as LiF, Li_2_O) and organic lithium compounds during cycling [38–41]. This thick and chemically unstable SEI significantly aggravates electrode polarization and capacity degradation. Secondly, due to the insufficiency of mechanical restraint between the Si particles and the polymer adhesive, repeated stress causes the loss of adhesion between adhesive and particles, resulting in the pulverization and delamination of the electrode structure. In addition, the interface between Si and the conductive additive forms a local region of conductive network failure due to particle migration, rearrangement, and electrode expansion. Therefore, the synergistic regulation of interfacial stability is the key to improving the long-cycle performance of Si anodes.

The deep mechanism of interfacial collapse can be traced back to the inherent volume expansion property of Si materials. Theoretical calculations indicate that the migration of lithium ions along the < 110 > direction in Si crystals has the lowest energy barrier, and this direction corresponds to the most significant lattice expansion [42]. The (220) crystal plane is precisely the characteristic diffraction plane of the < 110 > direction in the XRD spectrum. Therefore, by monitoring the change in the spacing of the (220) crystal plane, the expansion behavior along the < 110 > direction can be quantitatively tracked. Ideally, anisotropic expansion should cause the spacing of the (220) crystal plane to fully recover after lithium removal, thereby achieving reversible structural evolution. However, for traditional SiNPs, in situ X-ray diffraction (XRD) analysis shows that the spacing of the (220) crystal plane does not recover after lithium removal, indicating irreversible structural damage. This irreversibility stems from the isotropic volume expansion during lithiation, resulting in microcracks and lattice distortions within the particles, which in turn disrupts the structural integrity of the electrode.

Additionally, the inherent lattice distortion also causes stress concentration at the interface scale. When microcracks exist on the surface of Si particles, the local lithium-ion flux significantly increases due to reduced interfacial impedance, causing excessive expansion in this area and subsequently exerting pressure on the SEI layer. As shown in Fig. 2b, quantitative analysis based on an electrochemical-mechanical coupling model reveals this stress evolution process [43]. The model presets geometric defects of varying depths on the SEI surface to simulate actual structural inhomogeneity. During discharge, the Von Mises stress gradually accumulates at the defect center. When the stress reaches the SEI yield strength (approximately 10 MPa, equivalent to 1% of its elastic modulus), the SEI undergoes structural failure. Notably, smaller Si particles exhibit slower stress growth rates, making the SEI less prone to failure. This phenomenon is closely related to the reversibility of the (220) crystal plane: small-sized particles better maintain anisotropic expansion behavior, facilitating easier recovery of lattice distortion, which results in fewer surface defects and reduced SEI stress concentration. In situ atomic force microscopy (AFM) observed the rapid fragmentation of micro-sized Si (μSi) particles into submicron-sized particles during the first lithiation (Fig. 2c, d), accompanied by a volume expansion of 180% (Fig. 2e) [44]. During the delithiation process, the volume contraction triggered vertical cracks, and the exposed fresh surface initiated the continuous regeneration of SEI. However, the regeneration of SEI does not counteract the accumulation of mechanical stress, and the expansion of cracks ultimately leads to particle segregation.

These phenomena suggest that Si anode failure is a coupled chemo-mechanical dynamic process. Mechanical stress creates conditions for continuous and uncontrolled interface chemical reactions by triggering particle rupture and SEI damage. Meanwhile, the thick and uneven SEI generated by chemical side reaction further intensifies local stress concentration and electrode polarization. Therefore, the stability of the interface is not only limited by the chemical passivation ability of SEI, but also depends on its adaptability to mechanical stress. Solving the interface failure of Si anodes requires breaking through the single-dimensional control thinking and must simultaneously consider the chemical passivation of SEI, stress adaptation and charge transport optimization.

### Mechanical Instability and Structural Degradation

The interface stability of Si anodes faces significant challenges from mechanical instability and structural degradation. This issue stems from the significant volume change caused by the alloying reaction between Si and lithium during lithiation/delithiation. This periodic expansion and contraction of volume will generate significant mechanical stress within the active particles, which is the direct cause of interface structure damage [45, 46].

**Fig. 3:**
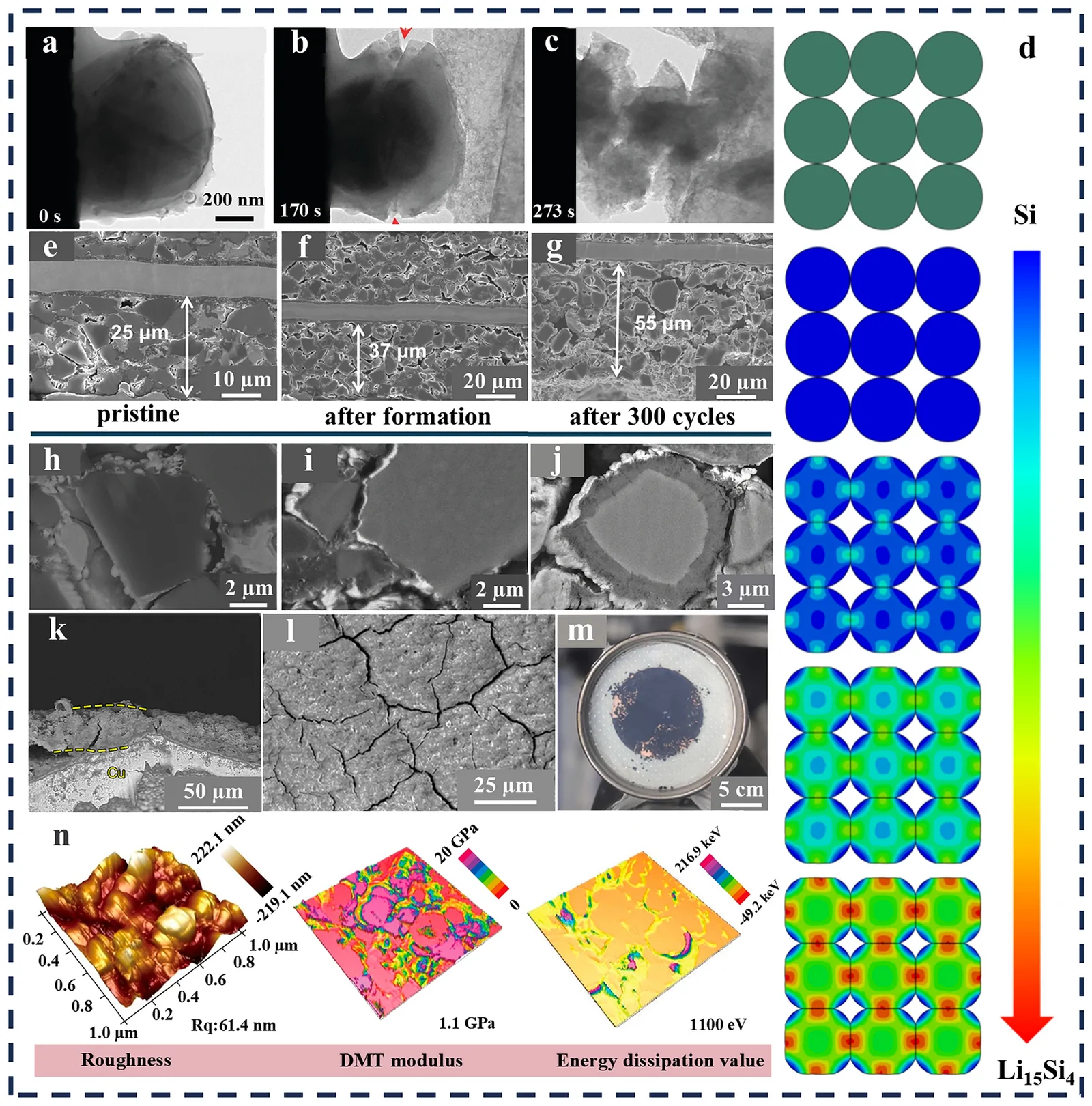
**a-c** In situ TEM image of surface cracking and fracture of SiNPs during lithiation process. Reproduced with permission [47]. Copyright, 2012 American Chemical Society. **d** Spatial distribution evolution of von Mises stress during the Li^+^ intercalation process. Reproduced with permission [48]. Copyright, 2025 American Chemical Society. **e–g** Cross-sectional images of Si electrodes after different cycling numbers and their corresponding magnified images **h-j**. Reproduced with permission [49]. Copyright, 2024 Royal Society of Chemistry. **k** Cross-sectional scanning electron microscopy (SEM) image of the Si electrode after cycling. Reproduced with permission [50]. Copyright, 2023 American Chemical Society. **l-m** SEM image and digital photograph of Si electrode after 80 cycles. Reproduced with permission [51]. Copyright, 2022 Elsevier. **n** Surface roughness, DMT modulus mapping, and dissipation mapping images of Si. Reproduced with permission [52]. Copyright, 2024 American Chemical Society

The origin of interface mechanical failure is closely related to the stress concentration caused by uneven volume changes, especially at the interface between crystalline Si and amorphous Li_x_Si alloy. The results of in situ transmission electron microscopy (TEM) (Fig. 3a–c) indicate that when the Si particle size exceeds the critical value (approximately 150 nm), the circumferential stress on particle surfaces will change from compressive stress to tensile stress, resulting in the crack nucleation and propagation, and ultimately leading to particle rupture [47]. In addition, this process is further visualized through finite element simulation (Fig. 3d) [48]. In the lithium-ion insertion process, von Mises stress is highly localized, with distinct stress concentration areas forming at the inter-particle gaps, which are consistent with the observations in the in situ experiments. This theoretical simulation confirms that the uneven volume changes are the fundamental cause of mechanical failure.

Furthermore, repeated volume changes can also lead to profound degradation of the interface. For micro-sized Si particles, their relatively low specific surface area can slow down the initial formation of the SEI, but the more intense volume expansion leads to severe particle pulverization, electrode cracking, and delamination. Cross-sectional SEM images of the Si electrode at different cycling stages (Fig. 3e–g) clearly reveal the gradual deterioration of the electrode structure: the electrode thickness continuously expands from the original 25 μm, increasing to 46 μm after 10 cycles, and reaching 55 μm after 300 cycles, indicating a volume expansion of up to 184% within the first 10 cycles [49]. Correspondingly, the average size of the Si particles gradually increases from 3.03 to 4.04 μm after 300 cycles, and the particle stacking becomes loose, with numerous voids appearing between particles. This not only disrupts the electrical contact between active particles but also hinders rapid charge transfer to the current collector. This structural degradation severely impedes charge transport and damage of electrode mechanical integrity.

It is worth noting that even in the absence of macroscopic cracks on individual particle surfaces, severe failure occurs at the interface. As shown in Fig. 3h–j, the surface of the Si particles undergoes continuous corrosion, forming a porous interface layer up to 2.5 μm thick. Energy-dispersive spectroscopy (EDS) analysis confirms this layer to be the SEI film formed by the accumulation of electrolyte decomposition products. Due to the inherently porous nature of the SEI layer, the electrolyte continuously penetrates and reacts with the underlying Si matrix, causing progressive thickening of the interface. Repeated volume expansion/contraction generate critical tensile stress on the Si particle surface, causing the outer layer of the particles to disintegrate into nanoscale fragments. These fragments, having lost connection to the conductive network, become detached from the Si core and are encapsulated within the continuously growing SEI matrix, forming a heterogeneous composite interphase. Raman spectroscopy analysis further reveals a characteristic peak at ~ 980 cm^−1^ at the inner interface near the Si matrix, corresponding to lithium silicates (Li_x_SiO_y_). This indicates that the detached Si fragments undergo side reactions with oxygen-containing species within the SEI (Li_2_O or decomposed carbonates), irreversibly consuming active lithium and active Si.

The most severe failure occurs when the electrode is unable to withstand repeated stress–strain cycles and ultimately releases energy forming cracks (Fig. 3k, l) [50, 51]. This creates isolated regions that disrupt ion and electron transport pathways, resulting in a significant amount of “dead Si.” Moreover, the initial dense structure of the electrode completely collapsed, with significant delamination occurring at the interface between the current collector and the active layer (Fig. 3m). More seriously, the cracks cause new active surface exposure, continuously decomposing electrolyte, and resulting in a catastrophic decline of battery performance. The essence of the structural degradation can be quantified with AFM by characterizing the microscopic mechanical properties of the electrodes. As shown in Fig. 3n, the Si electrodes with a traditional polyacrylic acid (PAA) binder exhibit a higher surface roughness and lower Derjaguin-Muller-Toporov (DMT) modulus after cycling, while the energy dissipation value is as high as 1100 eV [52].

In conclusion, mechanical instability and structural degradation form a cascading failure process across multiple scales. From the rupture of nanoparticles, to the loosening of the electrode microstructure, and finally to the cracking of the macroscopic electrode sheet, the core driving force of this process is the significant volume change effect. It is worth noting that there are significant differences in specific surface area and SEI formation behavior between nano-silicon and micro-silicon. The former has a higher specific surface area, which results in faster SEI growth, while the latter has a lower specific surface area but experiences more intense volume expansion. However, the interface failure of both ultimately originates from the chemo-mechanical coupling damage caused by volume changes. Therefore, any effective optimization strategy for Si anodes must take stress management and structural stability enhancement as its core goals. Through multi-scale collaborative design, this failure chain can be blocked, thereby fully leveraging the high-capacity advantage of Si anode materials.

### Complex/Unstable Solid-Electrolyte Interphase

The formation and evolution of SEI film on the Si surface is a critical, yet highly complex and unstable dynamic process. As is well known, this process begins before the first lithiation. The organic electrolyte components on the electrode surface (such as EC and FEC) undergo irreversible reduction decomposition, forming a complex solid-state electrolyte interphase layer [53–55]. SEI is considered to be mainly composed of three types of components: inorganic salts (such as Li_x_F_y_, LiF, Li_2_CO_3_), organic lithium compounds (such as ROCO_2_Li, RCO_2_Li), and polyethylene oxide (PEO) type polymers [56, 57].

Many previous studies have reported that the SEI formed in carbonate electrolytes exhibits a bilayer structure, consisting of a dense inner inorganic phase and a more porous outer organic phase. In recent years, advanced characterization techniques have made it possible to directly observe the near-intrinsic state of the SEI [58, 59]. For instance, cryo-electron microscopy observations revealed that after the first discharge of the Si anode, the surface SEI exhibited a mosaic structure where inorganic nanocrystals (such as Li_2_O and LiF) were embedded in an amorphous organic matrix. The components containing Li_2_O and organic carbonates formed during the lithiation process will be oxidized or react with Si during the delithiation process to generate amorphous lithium silicate (Li_x_SiO_y_) [60]. Advanced element tomography and cryogenic scanning transmission electron microscopy (cryo-STEM) analysis also show the SEI gradually grows toward Si particles as the cycling process continues, eventually forming a porous “plum-pudding” structure [34]. This further hinders the transmission of ions and electrons, leading to the formation of “dead” Li_x_Si alloys and accelerating the capacity degradation [58].

In addition, the low intrinsic electron conductivity of Si also has a profound impact on the formation process of the initial SEI. Research indicates that during the initial lithiation process, electrons must be transported from the current collector to the Si surface to drive the reduction and decomposition of electrolyte components. However, as the Si surface is covered by a natural oxide layer (SiO_x_) with poor conductivity, electrons tend to migrate along low-resistance paths such as defects, causing the SEI to nucleate and grow preferentially in regions accessible to electrons [61, 62]. As lithiation progresses, Si gradually transforms into Li_x_Si alloys with significantly enhanced electronic conductivity, and the reaction front migrates from the surface inwards. At this stage, if the initial SEI lacks sufficient electronic insulation, electrons may still reach the SEI/electrolyte interface via tunneling or defect sites, causing the SEI to continue thickening on top of the existing film rather than forming an ideal, dense passivation layer. This dynamic nucleation and non uniform growth, triggered by low electronic conductivity, makes it difficult for the SEI on the Si surface to establish a stable and effective passivation effect in the initial stage.

However, the long-term stability of SEI not only depends on its mechanical strength, but also relies on the chemical affinity between each component and the Si surface, that is, the strength of the interfacial bonding energy. Density functional theory (DFT) calculations show that the natural oxide layer typically covering the Si surface has a relatively high chemical affinity with certain inorganic components such as Li_2_O and Li_4_SiO_4_, which can form strong chemical bonds; however, common inorganic components such as Li_2_CO_3_ and LiF often have a weaker binding with the Si surface, mainly relying on physical adsorption [63, 64]. For organic components such as ROCO_2_Li and polyether-type oligomers, their interaction with the Si surface is more complex: on the one hand, the polar functional groups in their molecules can form hydrogen bonds with the hydroxyl groups on the SiO_x_ layer, showing a certain affinity; on the other hand, the organic framework of these components exhibits inherent incompatibility with the inorganic Si surface, which limits the strength of the interfacial bonding [65].

**Fig. 4:**
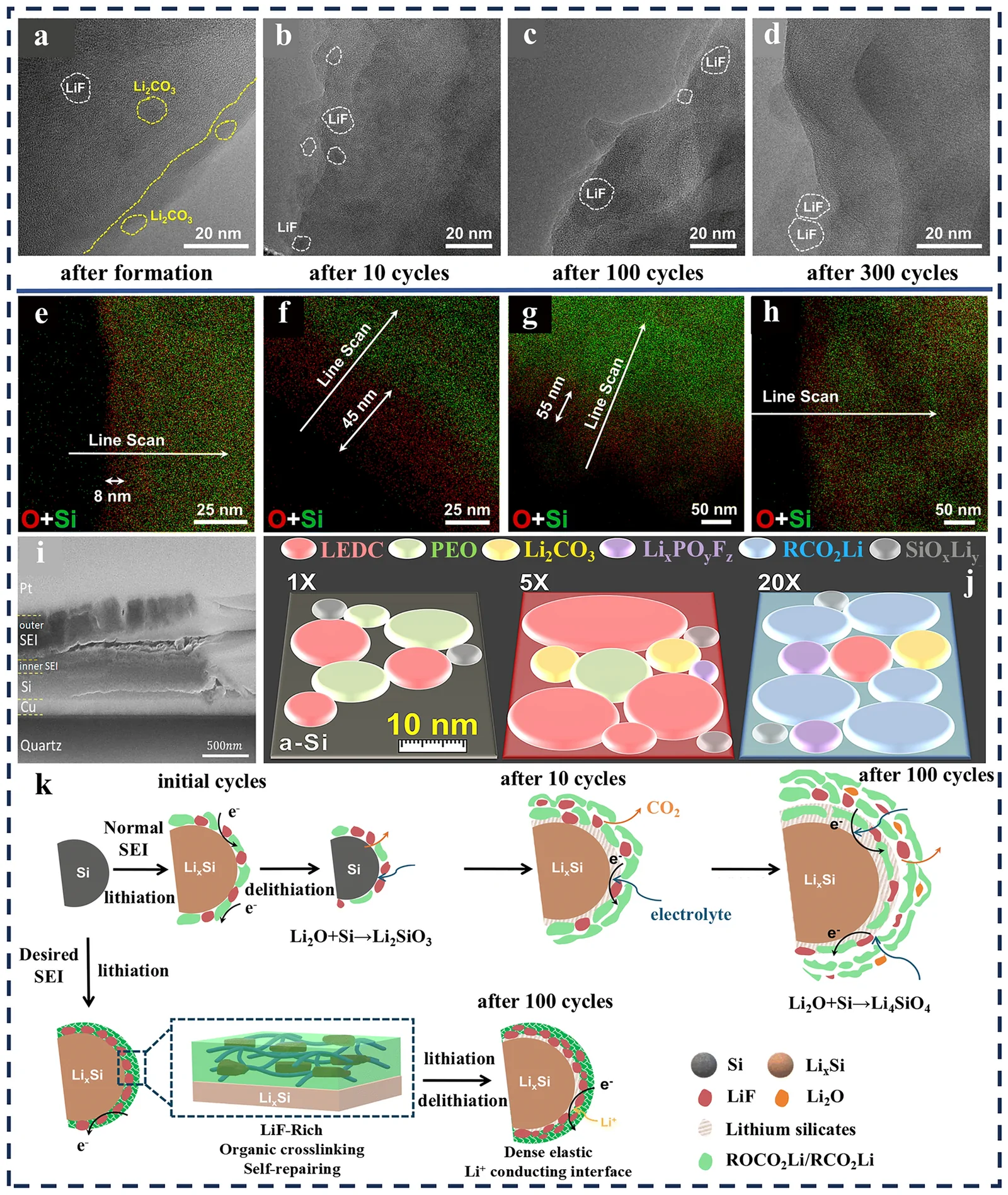
**a-d** HRTEM images of the SEI on the Si electrode after different cycle numbers, and the corresponding elemental distributions of the SEI layer **e–h**. Reproduced with permission [49]. Copyright, 2024 Royal Society of Chemistry. **i** Cross-sectional FIB images of Si electrodes after 5 cycles. Reproduced with permission [19]. Copyright, 2020 Elsevier. **j** Schematic of the evolution of SEI cross-sectional scans with increasing number of constant-current cycles. Reproduced with permission [66]. Copyright, 2019 Elsevier. **k** Schematic of SEI growth on Si anode. Reproduced with permission [49]. Copyright, 2024 Royal Society of Chemistry

It is noteworthy that the substantial volume change of Si anodes keeps this interface layer in continuously rupture and regeneration during lithium extraction process. This “breathing effect” not only irreversibly consumes the active lithium and the electrolyte, but also causes the SEI layer to significantly increase with the increase of cycles (Fig. 4a–h) [49]. During this process, distinct through-cracks appear in the SEI layer, while partial delamination occurs on the SEI out layer (Fig. 4i) [19]. This suggests that the chemical composition of SEI also evolves over time. As shown in Fig. 4j, after the initial cycle, the SEI mainly consists of lithium di-carboxylate (LEDC) and polyethylene oxide (PEO) type oligomers. As the cycle progresses, it gradually transforms into mainly containing carboxylate compounds (RCO_2_Li) and fluorine-containing species (Li_x_PO_y_F_z_), presenting a certain chemical gradient [66]. This evolution demonstrates that the SEI chemical composition continuously changes as the electrochemical process progresses instead of a static interface, which further increases its instability and unpredictability. However, it is also reported that the SEI rapidly grows during the initial cycling and may stabilize after a certain number of cycles, with organic fragments such as −OCH_2_CH_2_O− present within the SEI [67].

In summary, the SEI on Si anode is a dynamic interface, which constantly grows and reconfigures. As shown in Fig. 4k, its formation and evolution result from the combined action of the “bottom-up” (electrolyte penetration decomposition) and “top-down” (surface electrochemical reduction) mechanisms. The ultimately formed thick SEI exhibits complex chemical heterogeneity and a multi-layered structure. Therefore, it is crucial to establish an ideal SEI, which should possess excellent ionic conductivity to facilitate the transport, excellent electron insulation to prevent side reactions, and good mechanical elasticity to accommodate volume changes.

### Insufficient Interfacial Ion/Electron Transport

Despite the significant advantage of Si anodes in specific capacity, the slow ion and electron transport kinetics at their interfaces severely limit the rate performance, presenting another critical challenge for practical application. To enable fast charging, interfacial charge transport must be improved. The essence of this challenge involves multiple interrelated constraints, including the intrinsic properties of the material, the dynamic evolution of the interface, and solvation structures [68, 69].

**Fig. 5:**
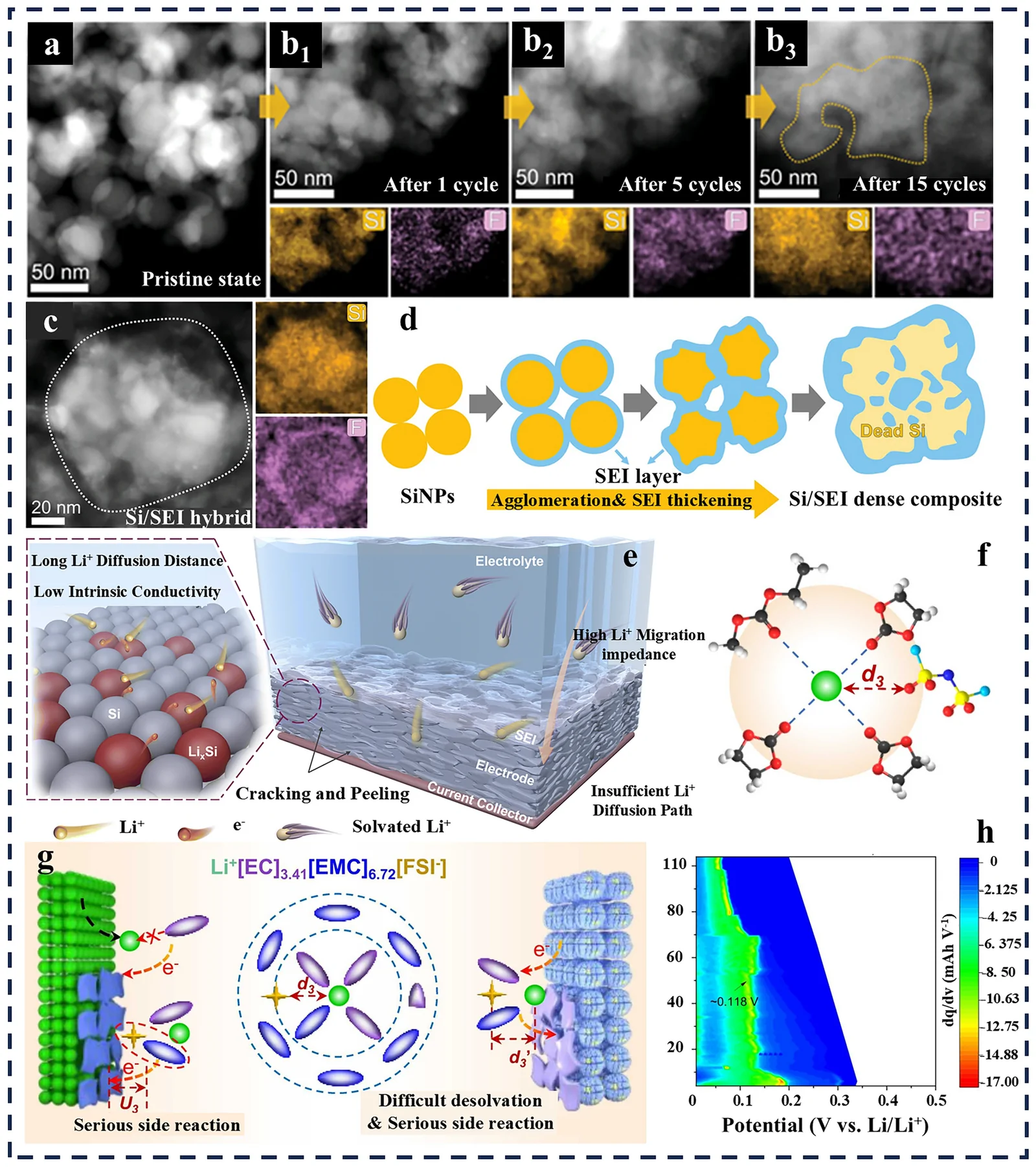
**a** HAADF-STEM image of the SiNPs before the cycle. **b, c** HAADF-STEM images and corresponding EDX maps of SiNPs during the cycling process. **d** Schematic diagram of the structural degradation mechanism of SiNPs. Reproduced with permission [35]. Copyright 2023, John Wiley and Sons. **e** Schematic of limiting factors for the poor kinetic performance of Si anodes. Reproduced with permission [70]. Copyright, 2025 Royal Society of Chemistry. **f** Schematic of the coordination structure of Li^+^-solvents and Li^+^-FSI^−^ pairs in the solvation structure. **g** Solvation structure and interfacial model of 1.0 M LiFSi in EC/EMC electrolyte. **h** Differential capacity (dQ/dV) of the Si lithiation. Reproduced with permission [71]. Copyright, 2024 Elsevier

Firstly, Si, as a typical semiconductor material, has an electron conductivity much lower than that of metals or graphite-type anodes. The low intrinsic electron conductivity (10^−5^ ~ 10^−3^ S cm^−1^) and the low Li^+^ diffusion coefficient (10^−14^ ~ 10^−13^ cm^2^ S^−1^) constitute the inherent limitations of charge transport. Secondly, during cycling, SiNPs undergo agglomeration while SEI components permeate between the particles, forming a dense Si/SEI composite structure (Fig. 5a–c). The thickened SEI not only hinders the electron conduction between the particles due to its electronic insulating properties, but also significantly lengthens the path for lithium ions to migrate from electrolyte to Si particles. Moreover, the aggregates further increase the effective distance for charge transfer, leading to the “deactivation” of a large amount of dead Si, thus causing rapid capacity degradation (Fig. 5d) [35]. Therefore, the limiting reaction kinetics factors span multiple scales. The intrinsic defects in the material and excessive growth of the SEI collectively form a “dual physical barrier” that directly disrupts charge transport pathways (Fig. 5e) [70].

Furthermore, the solvation structure of the electrolyte influences the interfacial ion migration energy barrier. In carbonate-based electrolytes, the strong coordination interaction between the anions (FSI^−^) and Li^+^ brings them closer to the Li^+^ center. However, its poor reduction stability leads to its preferential decomposition on the Si anode surface, resulting in an unstable SEI with abundant organic components and exhibiting nonuniform ion conductivity (Fig. 5f, g), further leading to a high barrier for ion migration at the interface. The differential capacity curve also reveals a rapid decay in the intensity of the lithiation potential, and the polarization potential shifts to a lower value (Fig. 5h). This also indicates that the interface side reactions and the growth of SEI have caused severe polarization and slow reaction kinetics [71].

In summary, the intrinsic electronic conductivity and low ionic conductivity of Si constitute the inherent bottleneck for charge transport. The uncontrollable thickening of the SEI during cycling and the agglomeration of particles significantly extend the ion/electron transport paths and increase the interface impedance. Meanwhile, the thermodynamic instability of the electrolyte and its unsatisfactory solvation structure chemically determine the formation of the high-resistance interface phase. These three factors, respectively, from the aspects of the bulk phase, physical structure and interface chemistry, jointly point to and exacerbate the transmission barrier at the interface, ultimately leading to a sharp decline in the kinetic performance of the Si anode.

## Engineering Multifunctional Interface via ALD/MLD

**Fig. 6:**
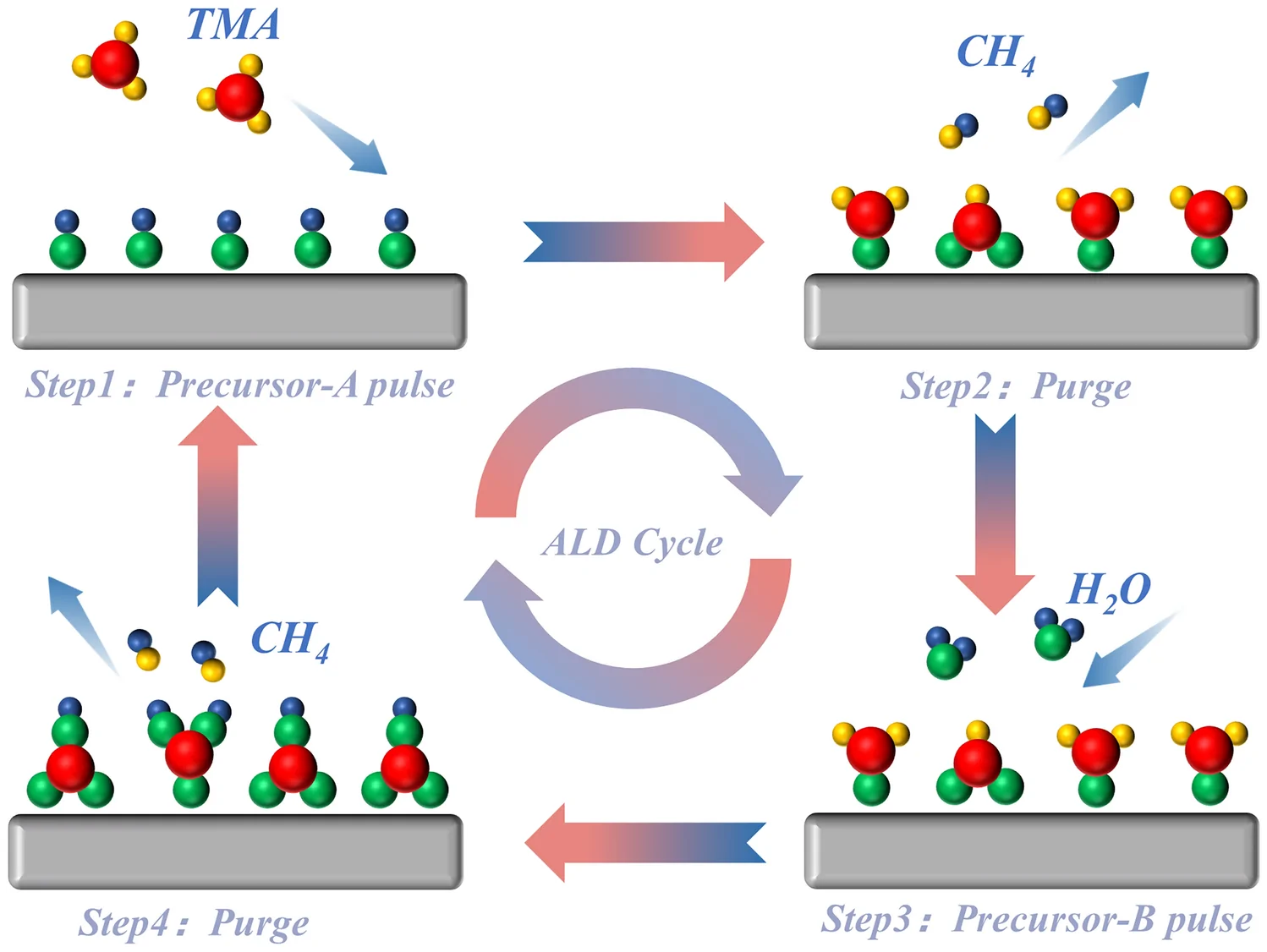
Schematic of the ALD process using TMA and H_2_O as precursors for depositing Al_2_O_3_

Since the introduction of ALD in the late 1970s [72], it has become a powerful technology for surface and interface engineering of related energy storage devices for its outstanding ability to control material deposition at the atomic level [73–77]. During the deposition process, two precursors are alternatively injected into the reaction chamber. And the chemical reaction of the new atomic-layer film is directly linked to the previous layer, which will construct deposition layer by layer (Fig. 6). ALD introduces only one precursor to the substrate surface at a time. This precursor reacts chemically with specific functional groups on the substrate surface until all active sites are occupied and the surface reaches saturation. At this point, even if more precursors are present, no further reactions occur [78, 79]. Based on this unique self-limiting characteristic, films deposited using ALD technology exhibit excellent uniformity, unmatched conformality, and consistent chemical composition [80]. So far, ALD technology has been capable to manufacture various inorganic materials, including metal oxides [81], nitrides [82], sulfides [83], fluorides [84] and complex compounds [85]. In recent years, ALD has shown great potential in the field of LIBs, mainly used in the design and fabrication of nanostructured components, customization of interfaces between components, and modification of active components [33, 86–89].

**Fig. 7:**
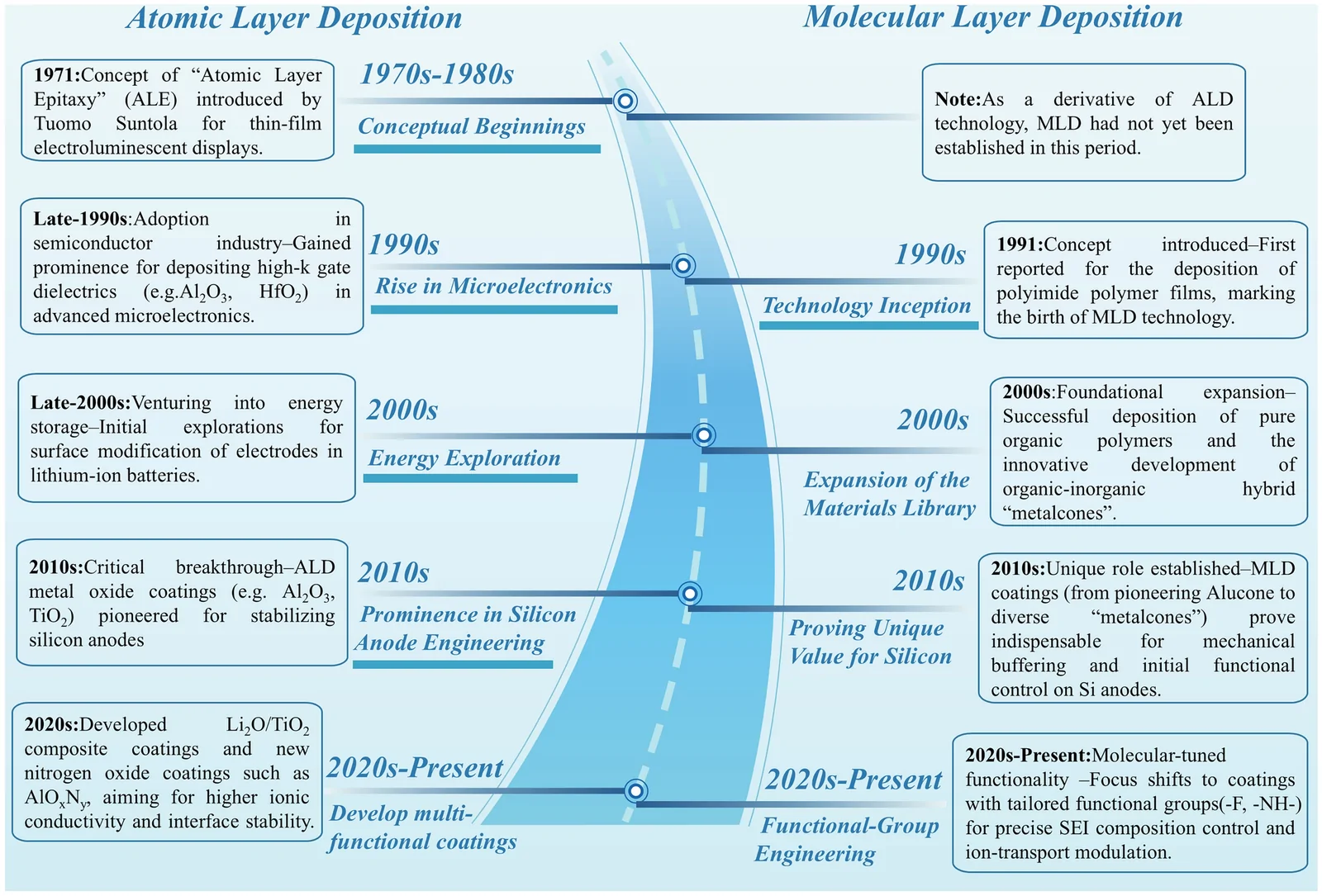
Historical development timeline of ALD and MLD technologies, highlighting key milestones from their inception to contemporary applications in Si anode interface engineering

As a derivative technology of ALD, MLD was designed to deposit polyimide polymer films from 1990s [90]. Similar to the principles of ALD, MLD can also precisely control the growth of materials at the atomic and molecular levels with high reproducibility (Fig. 7). MLD not only inherits the advantages of ALD in terms of uniformity and conformality, but also possesses excellent properties such as flexibility, low refractive index and low density [91, 92]. Moreover, MLD expands the range of precursor materials available. The deposited film materials encompass various organic polymers, including polyamide, polyurea, and polythiophene [93, 94]. By combining organic and inorganic precursors, MLD can also fabricate a wide variety of hybrid inorganic–organic films [95–98]. Although ALD and MLD follow the same self-limiting reaction principle, they exhibit significant differences in reaction pathways and growth kinetics [99]. ALD uses inorganic precursors and forms rigid inorganic coatings through ligand exchange or hydrolysis reactions, with the activation energy primarily originating from the cleavage of metal–ligand bonds [100]. MLD, on the other hand, uses organic or hybrid precursors (4-aminophenol, glycerol) to introduce an organic framework into the film. The reaction pathways are more complex, typically involving branched reaction chains where different functional groups exhibit distinct reactivities [101]. For example, in the deposition of Zincone using diethylzinc and 4-aminophenol, DFT calculations reveal that hydroxyl groups react preferentially over amino groups, and the removal of ethyl ligands as ethane is the rate-determining step [102].

The difference in reaction pathways and growth kinetics fundamentally determine the structural and chemical characteristics of the resulting coatings, which in turn directly influence the performance of Si anodes. The ALD reaction pathway is relatively simple, primarily because the inorganic precursors used (trimethylaluminum, titanium tetrachloride) typically contain only one metal center, with well-defined reaction sites (surface hydroxyl groups) and an absence of side reactions or branching pathways. This single-pathway, layer-by-layer growth mechanism renders the film formation process highly controllable and facilitates the formation of dense inorganic films, thereby providing effective mechanical constraint for Si anodes. In contrast, the MLD reaction pathway is more complex. On the one hand, MLD produces organic–inorganic hybrid films with intrinsic molecular flexibility through stepwise polymerization reactions. The incorporation of organic segments enables the coating to dissipate stress via rotation and bending of molecular chains, offering crucial mechanical buffering to accommodate the repeated volume changes of Si during cycling. On the other hand, the differential reactivity of functional groups in the precursors allows for the selective distribution of polar moieties within the film architecture. This reaction-kinetics-driven chemical tunability empowers MLD coatings to actively participate in lithium ion desolvation and the regulation of SEI composition.

Table 1 compares the core performance parameters of various thin film deposition techniques [103]. Among these, the most prominent advantage of ALD lies in its ability to achieve conformal coverage on high aspect ratio and complex three-dimensional structures. CVD can produce dense coatings, but its step coverage capability significantly decreases as structural complexity increases; while molecular beam epitaxy (MBE), pulsed layer deposition (PLD), evaporation, and sputtering are prone to shadow effects in high aspect ratio structures, resulting in insufficient coverage of recessed areas. For Si anodes, uneven coating means local exposure, and these areas become initiation sites for preferential SEI rupture and side reactions, ultimately leading to electrode failure. In addition, compared with evaporation and sputtering techniques, ALD/MLD can produce pinhole-free dense films, which is crucial for preventing electrolyte penetration and ensuring the long-term stability of the SEI on the Si anodes surface. The techniques further distinguish themselves in achieving atomically sharp interfaces and low surface roughness, which are essential for constructing multilayer heterostructures and precisely regulating interface properties. Although MBE also possesses excellent interface control capabilities, its stringent vacuum requirements and low deposition rate limit its large-scale application in Si anodes interface engineering. Notably, unlike CVD, which relies on high-temperature processes, and sputtering techniques, which may introduce surface defects, ALD/MLD employs mild processing conditions that avoid potential damage to the Si material structure.

**Table 1:** Properties of various thin films deposition techniques (Reproduced with permission [103]). Copyright 2017, Elsevier

Properties		Chemical vapor deposition (CVD)	Molecular beam epitaxy (MBE)			Pulsed layer deposition (PLD)	Atomic/Molecular Layer Deposition (ALD/MLD)	Sputtering	Thermal/e-beam evaporation
Deposition rate		Good	Fair			Good	Poor	Good	Good
Film density		Good	Good			Good	Good	Good	Fair
Lack of pin-holes		Good	Good			Fair	Good	Fair	Fair
Thickness uniformity		Good	Fair			Fair	Good	Good	Fair
Sharp dopant profile		Fair	Good			Varies	Good	Poor	Good
Step coverage		Varies	Poor			Poor	Good	Poor	Poor
Sharp interfaces		Fair	Good			Varies	Good	Poor	Good
Low substrate temperature		Varies	Good			Good	Good	Good	Good
Smooth interfaces		Varies	Good			Varies	Good	Varies	Good
No plasma damage		Varies	Good			Fair	Good	Poor	Good

It is true that ALD and MLD also have their own limitations. Their layer-by-layer growth nature inherently leads to relatively low deposition efficiency, stemming from the precursor pulses required for each cycle and the subsequent purging processes. However, for Si anodes surface engineering, sacrificing some deposition efficiency to obtain unparalleled conformality, interface quality, and low-temperature process compatibility is often a necessary trade-off. To address this issue, the improved technologies for powder ALD reactors (fluidized beds and rotating systems) are now capable of uniformly coating particles with high surface area, while also enhancing the utilization rate of the precursors and reducing the processing time [104]. It is worth noting that early ALD/MLD research in battery systems focused primarily on cathode materials, where the coatings functioned mainly as chemical passivation and surface stabilization layers with relatively low demands on mechanical properties. The advancement of powder ALD reactor technologies has further accelerated the application of ALD/MLD to the interfacial modification of micro-sized Si particles, which exhibit even greater volume expansion and thus higher interfacial strain. Compared with nano-sized Si, micro-sized Si offers greater commercial viability owing to its lower cost and higher tap density, yet its more severe volume expansion imposes far more stringent demands on the mechanical and chemical stability of the interfacial layer. As discussed in Sect. 2, the interfacial failure of Si anodes is fundamentally a chemo-mechanical coupled process. ALD/MLD, by virtue of its atomic/molecular-level thickness precision, ability to deposit conformal coatings on complex geometries, and capacity to tune interfacial mechanical properties across a continuum from rigid inorganics to flexible hybrid polymers, offers a uniquely precise means of addressing these interfacial challenges. This strong alignment between the interfacial demands of Si anodes and the unique capabilities of ALD/MLD has established the latter as an indispensable platform for Si anode interface engineering [105]. Relevant coating systems and electrochemical performance are summarized and presented in Table 2.

**Table 2:** Summary of various Si anodes deposited by ALD/MLD

Coatings	Size	Precursor	Thiknes (nm)	performance	Current (mA g^–1^)	ICE (%)	SEI Characteristics	Deposition Mode	Refs.
Al_2_O_3_	Nano	TMA+H_2_O	5	–	–	–	LiF-rich	Electrode	[112]
Al_2_O_3_	Micro		2	1100 mAh/g after 100 cycles	3750	>83.2	–	Electrode	[113]
Al_2_O_3_	Nano		1.1	99.89% after 40 cycles	420	–	LiAlO_2_	Electrode	[114]
Al_2_O_3_	Nano		8	827.3 mAh/g after 100 cycles	50	–	–	Electrode	[115]
Al_2_O_3_	Micro		3	–	–	–	Thin SEI	Electrode	[116]
TiO_2_	Nano	TTIP+H_2_O	10	1600 mAh/g after 100 cycles	359	91	Thin SEI, LiF	Electrode	[119]
TiO_2_	Nano	TTIP+O_2_	1.5	1700 mAh/g after 200 cycles	718	89	Suppressed Li_2_CO_3_	Electrode	[120]
TiO_2_	Nano	TTIP+H_2_O	3	1583mAh/g after 50 cycles	420	80.82	-	Particle	[121]
TiO_2_	Nano	TTIP+H_2_O	-	1156 mAh/g after 500 cycles	600	65	-	Particle	[129]
ZnO	Nano	DEZ+H_2_O	3	>1700mAh/g after 160 cycles	840	63	Thin SEI	Electrode	[123]
ZnO	Nano	DEZ+H_2_O	20	846 mAh/g after 200 cycles	500	76.8	Uniform, condensed	Particle	[130]
HfO_2_	Nano	TDMAH+H_2_O	2.51	2020 mAh/g after 100 cycles	1200	79.7	Thin SEI, LiF-rich	Electrode	[124]
NiO	Nano	Ni(dmamp)_2_+O_3_	16	2002 mAh/g after 1050 cycles	700	78.6	Stable SEI, (~35 )	Particle	[125]
Li_2_O+TiO_2_	Nano	LiOBu/TDMAT	5	1300 mAh/g after 1150 cycles	2000	90.9	LiF-rich	Particle	[150]
TiN	Nano	TiCl_4_+N_2_	5	740 mAh/g after 80 cycles	17950	90.0	Thin SEI, LiF	Electrode	[165]
AlO_x_N_y_	Nano	TMA+N_2_/H_2_	2	1297 mAh/g after 140 cycles	359	93.9	Li_3_N/Li-Al-O	Electrode	[167]
Alucone	Nano	TMA+Glycerol	5	900 mAh/g after 150 cycles	358	-	Passivating layer	Electrode	[134]
Alucone	Nano	TMA+Glycerol	2.5	1490 mAh/g after 500 cycles	716	-	Passivating layer	Electrode	[135]
AlFHQ	Nano	TMA+FHQ	5	862 mAh/g after 200 cycles	2000	-	LiF-rich (20.6 at%)	Electrode	[147]
Zincone	Nano	TMA+HQ	3	1011 mAh/g after 200 cycles	1000	81.9	LiF-rich, thin SEI	Electrode	[146]
Titanicone	Nano	TiCl_4_+HQ	8	957 mAh/g after 450 cycles	1000	-	Thin SEI (~5 nm), stable	Electrode	[187]
Niobicone	Nano	Nb(OEt)_5_+HQ	17.7	689 mAh/g after 800 cycles	1000	91.2	LiF-rich (40 nm)	Electrode	[138]
Polyurea	Nano	ED+PDIC	3	1010 mAh/g after 1000 cycles	800	82.3	LiF-rich, Li_3_N	Electrode	[51]
Polyamide	Nano	MPD+TMC	0.5	> 1400 mAh/g after 150 cycles	358	81.9	-	Electrode	[140]
LiO_2_+Lithicone	Nano	LiOtBu+HQ	5	646 mAh/g after 850 cycles	2000	91.2	LiF-rich	Electrode	[50]
Zincone+TiO_2_	Nano	DEZ/TiCl_4_+HQ	15	753 mAh/g after 1000 cycles	2000	81.9	Inorganic-rich (<5 nm)	Electrode	[143]
Si+LiFHQ	Nano	Si_2_H_6_+SiCl_4_	21.1	1024 mAh/g after 1000 cycles	2000	73.8	Thin SEI, LiF-rich	Electrode	[171]

### Suppressing Interface Mechanical Failure

Repeated volume expansion and contraction is the main factor causing mechanical failure and capacity degradation. Conventionally, the strategy to mitigate this issue has relied on carbon coatings to buffer volume changes and maintain structural integrity. Ryu et al. demonstrated that uniform carbon coatings applied on two-dimensional Si nanosheets form a unique rippling morphology during delithiation, effectively releasing cycling-induced stress and significantly enhancing the structural stability of the electrode [106]. This work reveals the great potential of regulating mechanical stress through interface engineering. ALD/MLD, with its diverse precursor selectivity and designability, enables the deposition of coatings with tailored physical and chemical properties, effectively suppressing mechanical failure at the Si anodes interface through mechanical buffering and structural support [107–111].

**Fig. 8:**
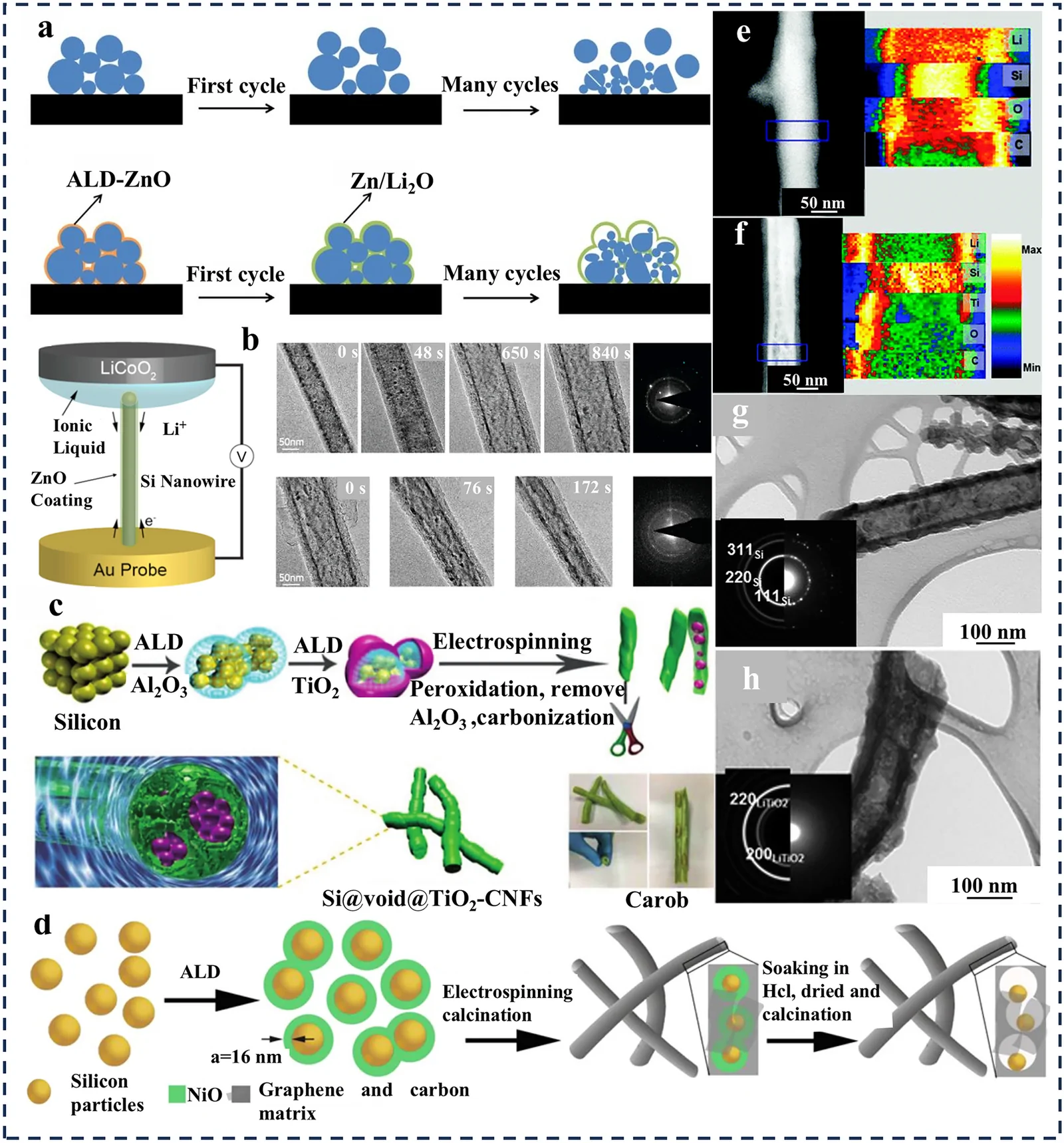
**a** Schematic of cycling of SiNPs electrode and electrode coated with ZnO. **b** Schematic of in situ TEM setup and SiNWs-coated of ZnO during lithiation and delithiation. Reproduced with permission [123]. Copyright, 2015 Elsevier. **c** Schematic of the synthesis process of Si@void@TiO_2_-CNF. Reproduced with permission [129]. Copyright, 2019 Royal Society of Chemistry. **d** Schematic of the composite electrode prepared by SiNPs after ALD deposition of NiO. Reproduced with permission [125]. Copyright, 2016 American Chemical Society. HAADF images and EELS element maps of the electrodes after 100 cycles: **e** SiNWs **f** SiNWs coated with TiO_2_. Reproduced with permission [119]. Copyright, 2013 Royal Society of Chemistry. **g, h** TEM micrograph of TiO_2_-coated SiNTs and after 100 cycles. Reproduced with permission [120]. Copyright, 2014 Royal Society of Chemistry

Specifically, rigid inorganic coatings, such as those based on Al_2_O_3_ [112–117], TiO_2_ [118–122], ZnO [123], HfO_2_ [124], NiO [125], SnO [126], function primarily by alleviating the volume expansion of Si and preventing the collapse of the electrode structure. For instance, Zhu and coworkers demonstrated that the ZnO coating on the SiNPs electrodes could be completely transformed into a conductive Zn/Li_2_O layer after lithiation [123]. This transformation occurred without cracking despite the expansion and contraction of the Si core, thereby structurally suppressing electrode pulverization (Fig. 8a, b). Similarly, TiO_2_ coatings are also widely studied to suppress Si electrode structure degradation for their excellent stability and mechanical strength [127, 128]. As shown in Fig. 8e, the surface of the uncoated Si nanowires (SiNWs) is completely covered by Li_2_CO_3_ after cycling [119]. In contrast, the SiNWs with TiO_2_ coating (Fig. 8f) exhibit a thinner SEI layer. Even after 100 cycles, LiTiO_2_ phase can still be observed. This indicates that the TiO_2_ coating significantly enhances the mechanical integrity of the electrode, and slows down the thickening of the SEI. To further enhance the protection, the research has expanded from simple surface coating to three-dimensional structure design. For instance, TiO_2_ coatings are constructed on both the inner and outer surfaces of Si nanotubes (SiNTs) to improve structural stability during cycling [120]. Figure 8g reveals that the TiO_2_ coating prepared by ALD has a uniform amorphous structure, providing an initial stable interface for the electrode. After cycling, the inner and outer coatings of the double-sided TiO_2_-coated SiNTs (Fig. 8h) transform into the crystalline LiTiO_2_ phase. Its high electronic conductivity effectively optimizes the electrode’s rate performance while maintaining structural integrity.

Nevertheless, standalone rigid inorganic coatings remain prone to fracture when mitigating extreme volume changes, especially over long-term cycling. ALD technique is employed to design more sophisticated three-dimensional structures. For example, a composite electrode inspired by the structure of the acacia pod was proposed [129]. Firstly, Al_2_O_3_ and TiO_2_ layers were successively deposited on the SiNPs surface. Subsequently, a Si@void@TiO_2_-CNF composite material was prepared by combining electrospinning and carbonization processes (Fig. 8c). This design reserves cavities for the expansion and contraction of the Si core, and the elastic carbon fiber matrix effectively disperses stress, achieving the ultimate suppression of mechanical failure at the structural level. Furthermore, the researchers elevated ALD from a surface modification tool to a means of creating three-dimensional space [107, 130, 131]. As shown in Fig. 8d, a novel electrode with a precisely eccentric hollow structure was in situ fabricated by depositing a NiO template layer on SiNPs via ALD, forming a composite with a carbon matrix [125]. The predefined void-to-Si-particle volume ratio of up to 3.4 in this design provides quantitative buffer space for volumetric expansion. This enables the carbon matrix to actively accommodate Si deformation, fundamentally resolving mechanical stress issues.

**Fig. 9:**
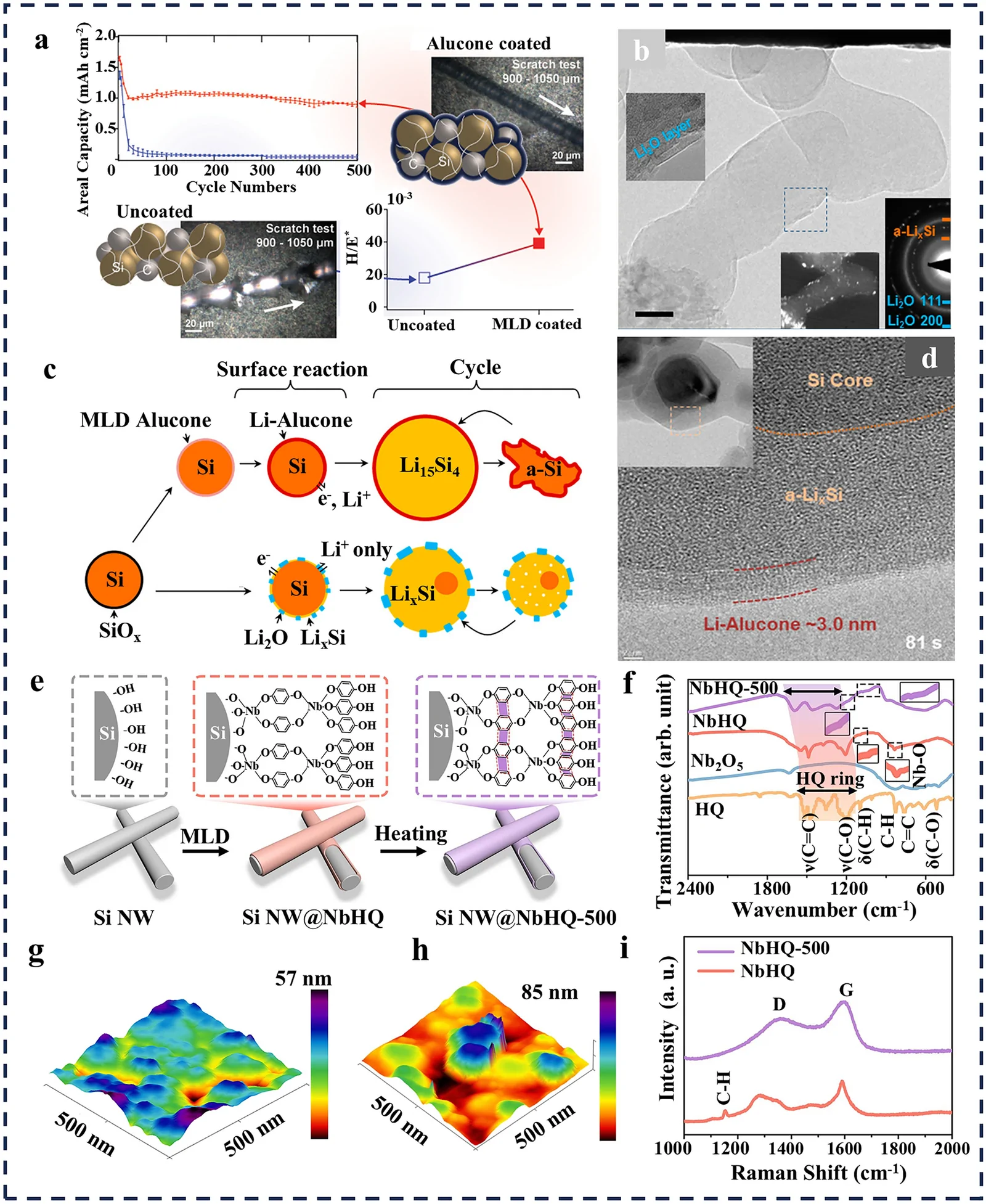
**a** Comparison of scratch test, electrochemical properties of uncoated Si and Alucone-coated Si electrodes. Reproduced with permission [135]. Copyright, 2017 American Chemical Society. **b** In situ TEM image of SiNPs coated with natural Li_2_O. **c** Schematic of the surface reaction of SiNPs for the uncoated and the Alucone-coated Si electrode. **d** In situ HRTEM image of Alucone-coated SiNPs after lithiation. Reproduced with permission [136]. Copyright, 2014 American Chemical Society. **e** Schematic of the annealing process after deposition of Niobicone on SiNWs. **f** FTIR spectra of HQ, Nb_2_O_5_, Niobicone and annealed Niobicone. **g, h** AFM images of Niobicone before and after annealing. **i** Raman spectra of Niobicone and annealed Niobicone. Reproduced with permission [138]. Copyright, 2024 Elsevier

Compared with rigid inorganic coatings, the inorganic–organic hybrid films prepared by MLD technology exhibit superior mechanical flexibility such as Alucone, Zincone [132, 133]. Its advantage lies in the fact that through the rotation and bending of the molecular chains, it absorbs strain energy, achieving a more uniform stress distribution and avoiding the catastrophic cracking caused by the common stress concentration in rigid coatings (key mechanical performance parameters are summarized in Table 3). Alucone coating can not only adapt to the repeated volume fluctuations of Si during cycling, but also maintain the electrode structure [134]. Figure 9a presents a comparison of scratch tests, electrochemical performance, and mechanical properties of the electrodes [135]. A series of mechanical characterizations have confirmed the enhancement of mechanical properties of the coated electrodes. Additionally, this coating can effectively remove the natural oxide layer on the Si surface during the deposition process [136]. For unmodified Si particles, the surface SiO_x_ will transform into discrete distributed Li_2_O crystal islands during the first lithiation (Fig. 9b). These insulating crystals hinder electron transmission and cause local current concentration, resulting in incomplete lithiation and irreversible capacity degradation [137]. The Alucone coating fundamentally enhances the cycling stability of the electrode by removing oxides and establishing an ionic/electronic hybrid conductive interface (Fig. 9c, d). It avoids the uneven lithiation and local stress concentration achieving uniform lithiation and further alleviating mechanical stress, which is difficult to achieve for many physical deposition coatings. To further optimize the mechanical properties of this flexible coating, post-processing techniques are introduced. For instance, the niobium-based “Niobicone” film can be crosslinked by annealing after deposition (Fig. 9e, f) [138]. This cross-linked structure significantly enhanced its mechanical strength and chemical stability, which is consistent with the film structure measured by AFM (Fig. 9g, h) and the increased I_D_/I_G_ value in the Raman spectrum (Fig. 9i) [139]. Therefore, this post-processing strategy ingeniously strikes a balance between “flexibility” and “strength”, by introducing cross-linking points to enhance the modulus while preserving the overall toughness of the polymer network, providing a new approach for customizing coating performance.

**Table 3:** Summary of mechanical properties and associated performance enhancements for representative MLD-derived coatings on Si anodes

Coatings	Young’s/Elastic Modulus (GPa)	Key Mechanical Finding/Indirect Metric	Characterization Method(s)	Refs.
Alucone	Increase>125%	Elastic reversibility: Increase>150%	Nano indentation, H/E* Calculation	[135]
AlFHQ	25.0	Electrode thickness after cycling: 135 µm vs. 181 µm (bare)	Nano indentation, Cross-Chapteral SEM	[147]
Zincone	-	The rate of electrode thickness change: 66% vs. 133%(bare)	Cross-Chapteral SEM	[146]
Titanicone	21±2	Volume expansion rate: 58% vs. 380%(bare)	Cross-Chapteral SEM, finite element	[187]
Niobicone	12.8 vs. 44.2(annealed)	After annealing through cross-linking reaction, the fracture toughness of Niobicone significantly improved.	AFM,FTIR, Ramanspectroscopy	[138]
Polyurea	SEI: 3.0 vs.1.5(bare)	Adhesion strength increased by 102.7%	AFM,universal peeling test	[51]
Polyamide	4.90±1.01	Hardness 0.12 ± 0.03 GPa (up by 50%)	Nano indentation	[140]
LiO_2_+Lithicone	48	Volume expansion rate: 42.2% vs. 89.2%(LiO_2_)	Nano indentation, Cross-Chapteral SEM	[50]
Zincone+TiO_2_	53	Volume expansion rate: 59.3% vs. 162.9%(bare)	Nano indentation, Cross-Chapteral SEM	[143]
Si+LiFHQ	-	Volume expansion rate: 3.25%% vs. 36.6%%(bare)	Nano indentation	[171]

**Fig. 10:**
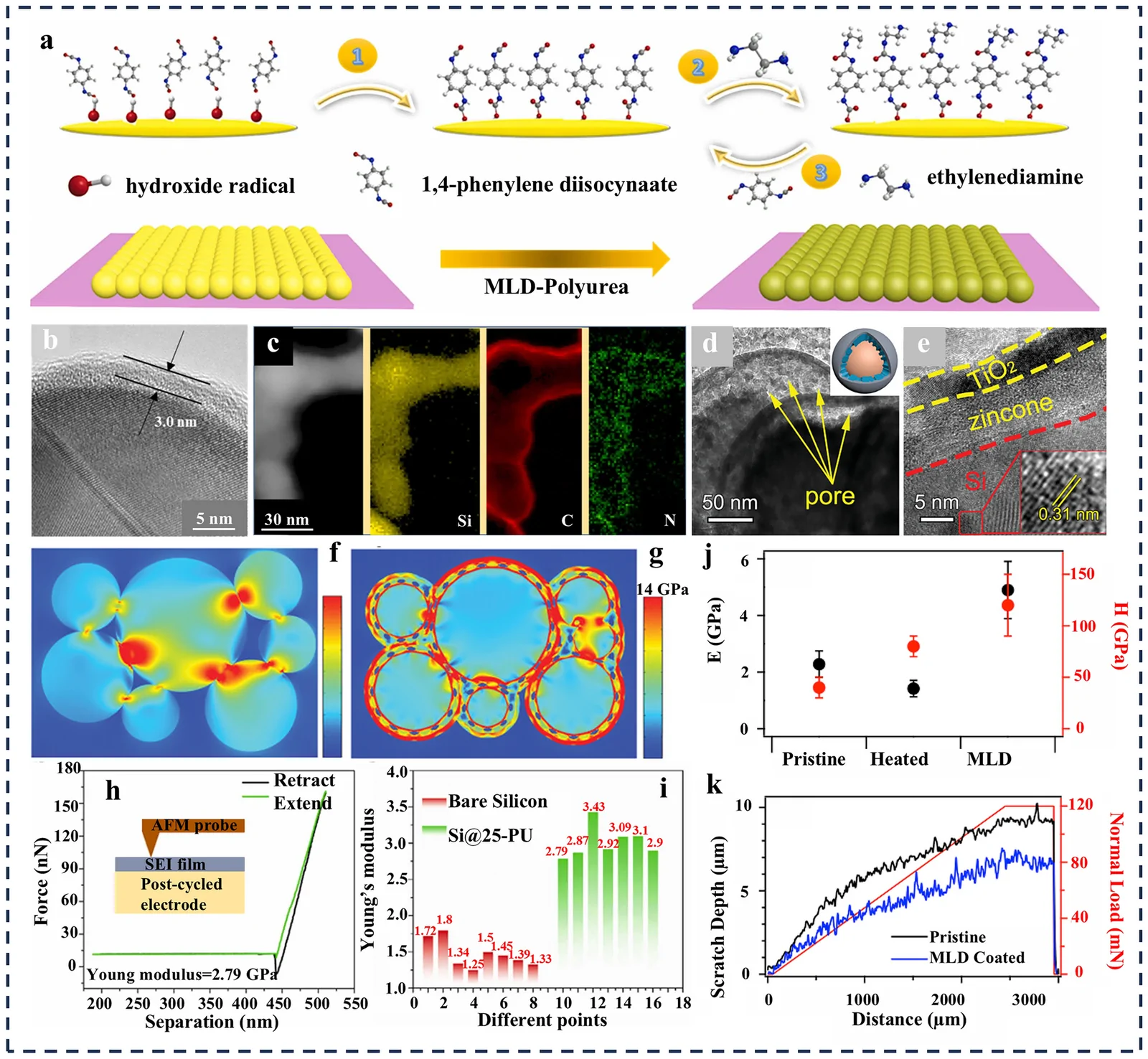
**a** Schematic of polyurea coating deposited on Si electrode. **b** HRTEM image of polyurea coatings. **c** STEM-EELS elemental mapping of Si@25-PU. Reproduced with permission [51]. Copyright, 2022 Elsevier. **d, e** TEM images of Si@zincone/TiO_2_. Stress distribution: **f** Si and **g** Si@zincone/TiO_2_. Reproduced with permission [143]. Copyright 2021, John Wiley and Sons. **h** Force–displacement curves of the Si@25-PU electrode after 80 cycles. **i** Young's modulus statistic of the pristine Si and the Si@25-PU. Reproduced with permission [51]. Copyright, 2022 Elsevier. **j** Elastic modulus and hardness values of the original electrode and the polyamide-coated electrode. **k** Scratch test of the pristine and polyamide-coated electrodes. Reproduced with permission [140]. Copyright, 2019 American Chemical Society

Certain inorganic–organic “metalcone” polymer interfaces can effectively alleviate the volume expansion and enhance cycling stability of Si-based anodes through the synergistic optimization of mechanical flexibility and interface chemistry. However, another polymer coating system with inherent flexibility, can also achieve more efficient stress dissipation and interface stability by providing abundant functional groups. For example, the polyurea coating formed on SiNPs surfaces via MLD (Fig. 10a–c) features an extensive internal hydrogen-bond network that not only ensures strong adhesion to the Si substrate but also provides pathways for lithium-ion transport [51]. More importantly, as demonstrated by the mechanical test data in Fig. 10h, i, this coating induces the formation of a LiF-rich SEI with higher modulus and more uniform distribution. Similarly, the aromatic polyamide coating has been proven to effectively address the issue of mechanical performance degradation caused by the decomposition of the electrode binder [140]. Through the spatial MLD technique, this coating forms a nano-scale, conformal, continuous three-dimensional polymer network within the electrode. Based on the inherent high strength and wear resistance of the aromatic polyamide, this network constitutes a stable mechanical framework. When polyacrylic acid (PAA) loses its adhesive function due to dehydration and forms polyacrylic anhydride, the polyamide framework can still maintain the structural integrity of the electrode. Moreover, the electrochemical inertness of the polyamide coating enables it to act as a protective barrier, providing stability to the electrode/electrolyte interface. This is particularly important because the decomposition products of PAA and the released water react with LiPF_6_ to form corrosive HF. However, this coating effectively inhibits the occurrence of side reactions by minimizing the direct contact between the active materials and the electrolyte. Nanoindentation and scratch tests (Fig. 10j–k) demonstrate that this coating significantly enhances the elastic modulus and hardness of the electrode. By strengthening the cohesion between particles, it effectively inhibits the peeling of active substances during cycling, thereby enhancing the mechanical stability at the macroscopic electrode level and improving the cycle life of the battery.

To overcome the limitations of single type coating system, cutting-edge research has shifted to design a “flexible inside and rigid outside” gradient functional interface combining the rigidity of ALD coating and the flexibility of MLD coating [141, 142]. For instance, the collaborative strategy proposed by Li et al. [143] involves two steps. Firstly, a flexible porous Zincone framework is deposited on the Si surface as a buffer layer to absorb internal stress. And then a rigid TiO_2_ film is connected on the Zincone to provide mechanical constraints and protection (Fig. 10d, e). Finite element simulations have shown (Fig. 10f, g) that this gradient structure can effectively disperse and homogenize the local stress during cycling, thereby fundamentally inhibiting the migration of active substances and structural pulverization.

In summary, ALD and MLD technologies offer powerful and precise toolkits for suppressing interfacial mechanical failure. The rigid inorganic coatings can offer a solid physical barrier, effectively constraining the initial expansion of Si through interface passivation and mechanical buffering. However, the rigid fracture still hinders the realization of high-rate performance and long cycle life. Inorganic–organic hybrid coatings are designed to improve this situation for their excellent flexibility, which can more efficiently buffer the cyclic stress through elastic deformation. The organic polymer coatings further stabilize the interface chemistry by their inherent flexibility and rich functional groups while dissipating stress. It is worth noting that the composite gradient structure that combines rigidity and flexibility ingeniously balances both instantaneous mechanical strength and long-term structural stability.

The remarkable effectiveness of these diverse strategies stems from the intrinsic alignment between the unique capabilities of ALD/MLD and the interfacial demands of Si anodes. This self-limiting, layer-by-layer growth approach ensures the formation of uniform and precisely controllable interface layers on Si anode materials of various architectures. This achievement remains challenging for traditional coating methods, as uniformity and thickness control heavily rely on the process. Issues such as the “coffee ring effect” during solvent evaporation or particle aggregation in the slurry often lead to incomplete coverage, local stress concentration, or excessive thickness, thereby hindering ion transport [144, 145]. More importantly, ALD/MLD enables the atomic/molecular-level design of graded mechanical properties and hybrid compositions. For example, constructing an “internally flexible, externally rigid” Si@zincone/TiO_2_ gradient structure requires sequential and precise deposition of different material layers, each with tailored modulus and interfacial bonding characteristics. This degree of structural precision and interfacial tailoring lies beyond the capability of conventional surface modification methods, where even uniform single-layer coatings remain a challenge.

### Regulating Interfacial Chemistry and SEI Evolution

The composition and stability of the SEI directly determine the cycle life and rate performance of Si anodes, as is well established. An ideal SEI should possess both ionic conductivity and mechanical stability, which can suppress the continuous decomposition of electrolytes. ALD/MLD technology precisely designs the interface chemistry of Si anodes, inducing in situ transformation and specific functional groups, achieving active control over the composition and evolution of SEI.

**Fig. 11:**
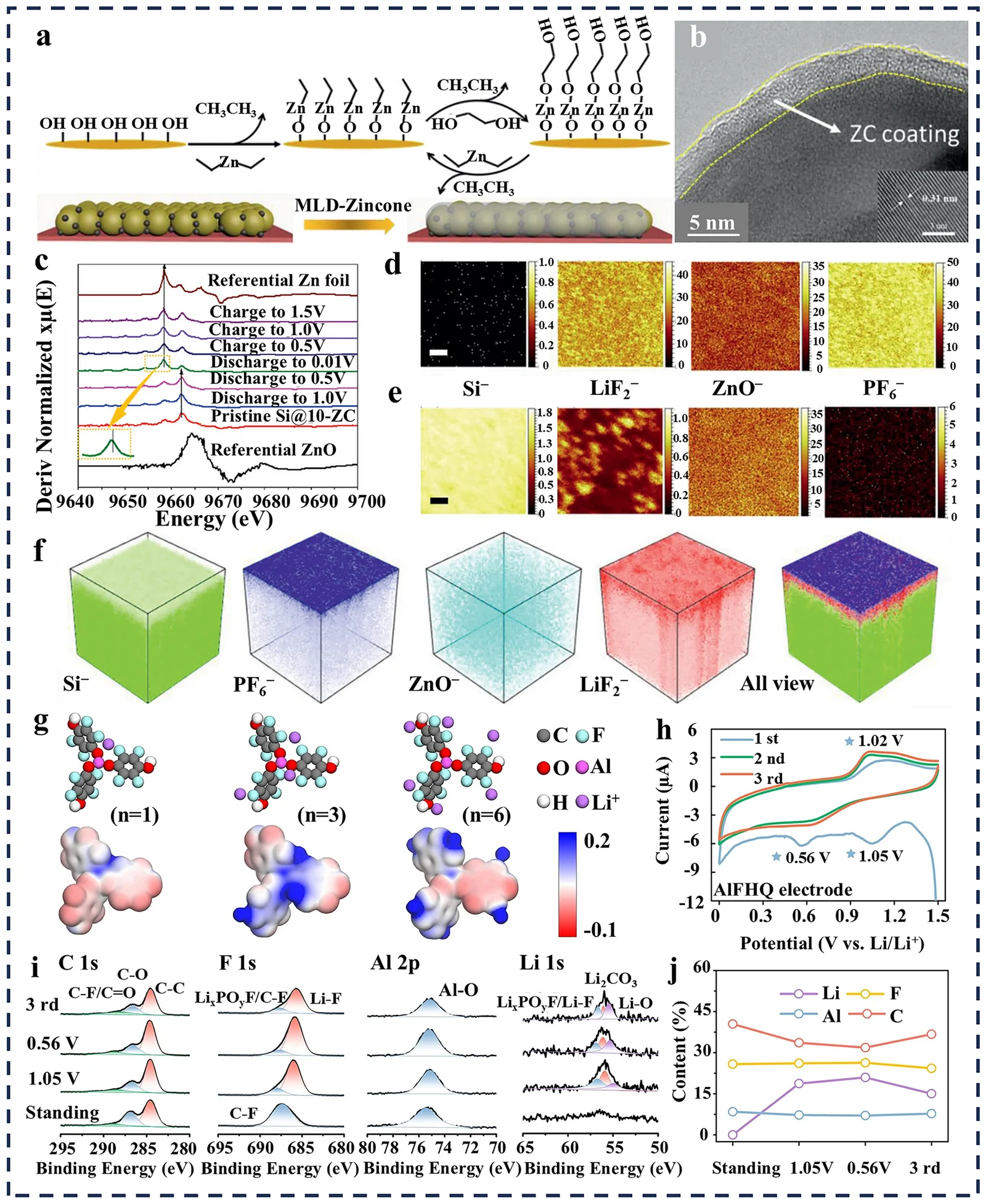
**a** Schematic of Zincone coating deposition on Si electrode. **b** HRTEM image of Zincone. **c** XAS spectral differential curves at different potentials. **d, e** TOF–SIMS images of Si@10-ZC electrode. **f** 3D reconstruction of secondary ion depth profiles obtained after 80 cycles. Reproduced with permission [146]. Copyright 2021, John Wiley and Sons. **g** Li^+^ adsorption configuration and corresponding ESP map of the AlFHQ. **h** CVcurves of AlFHQ film. **i** XPS characterization of AlFHQ film at different CV stages. **j** Composition of AlFHQ film at different CV stages. Reproduced with permission [147]. Copyright, 2025 Elsevier

The hybrid coatings prepared by MLD not only serve as physical barriers but also lay the foundation for stable SEI through their own electrochemical in situ conversion. As reported, the ultrathin Zincone (Fig. 11a–c) was partially reduced during cycling and formed a Li_x_Zn alloy with excellent electronic conductivity [146]. This transformed product, in conjunction with the following generated columnar-distributed LiF, jointly constructed an efficient ion/electron transport pathway. The depth analysis by Time-of-flight secondary ion mass spectrometry (TOF–SIMS) further confirmed that this coating facilitated the formation of thin and uniform SEI, with the spatial distribution of its key component LiF being optimized (Fig. 11d–f). Through the in situ transformation of the ultrathin coating and the regulation of SEI components, it provided a crucial interface support for the Si anode to achieve high cycling stability and rate performance.

Based on the in situ conversion concept, researchers have further advanced the design of precursors in the MLD process by directly introducing specific functional groups at the Si surface. The fluorine-rich aluminum-based hybrid (AlFHQ) coating embodies the design concept based on functionality [147]. DFT calculations (Fig. 11g) revealed that the strongly electronegative F atoms on the fluorinated benzene ring (–O–C_6_F_4_–) can effectively “pull” Li^+^ and promote its desolvation. Meanwhile, this coating also has excellent mechanical buffering ability, thereby achieving a synergistic improvement in both interface chemical and mechanical stability.

The CV curve on the AlFHQ film deposited on the copper foil (Fig. 11h) revealed that during the initial discharge, distinct reduction peaks were observed at 1.05 and 0.56 V, corresponding to the multi-step lithiation process of the Al–O–2,3,5,6-fluorobenzene framework. Notably, the peak at 0.56 V exhibited partial reversibility in subsequent cycles, with its potential shifting to 100 mV, while the peak at 1.05 V completely disappeared. Additionally, reversible oxidation–reduction peaks appeared and persisted at 0.65 and 1.02 V, mainly attributed to the reversible lithiation /delithiation process of the aromatic core species in the hybrid framework [148, 149]. This indicates that AlFHQ contains two different structural units: an irreversible fluorine-rich region that undergoes de-fluorination during the initial lithiation, and an aromatic nucleus fragment that contributes weak but persistent reversible oxidation–reduction activity. Ex situ XPS depth profiling (Fig. 11i) further confirmed this dual behavior. After cycling, the C 1*s* and Al 2*p* spectra remained almost unchanged, indicating that the Al–O–2,3,5,6-fluorobenzene structure maintained its structural integrity. However, the F 1*s* spectrum showed a clear transformation: the original C–F bond signal gradually weakened, while a strong LiF signal appeared. Figure 11j shows that the total fluorine content remained unchanged throughout the cycle, indicating that the fluorine atoms did not leach into the electrolyte but underwent a chemical state transformation from the covalent C–F bond to the ionic LiF in situ.

The AlFHQ coating functions through a dual mechanism of irreversible defluorination and reversible aromatic coordination. The irreversible defluorination delivers fluorine atoms to the interface, seeding the formation of a LiF-rich hybrid SEI with excellent mechanical properties. The reversible aromatic core species provides weak but persistent Li^+^ coordination sites, which may promote interface ion transport without significantly contributing to capacity. After the initial reconfiguration, the formed interface is composed of the stable Al–O–2,3,5,6-fluorobenzen structure embedded with LiF-rich, maintaining its structure and chemical stability during long cycles, inhibiting the continuous decomposition of the electrolyte and guiding uniform Li^+^ flux. The synergy of irreversible fluorine transfer and reversible aromatic coordination is the intrinsic mechanism for the synergistic improvement of interface chemistry and mechanical stability.

**Fig. 12:**
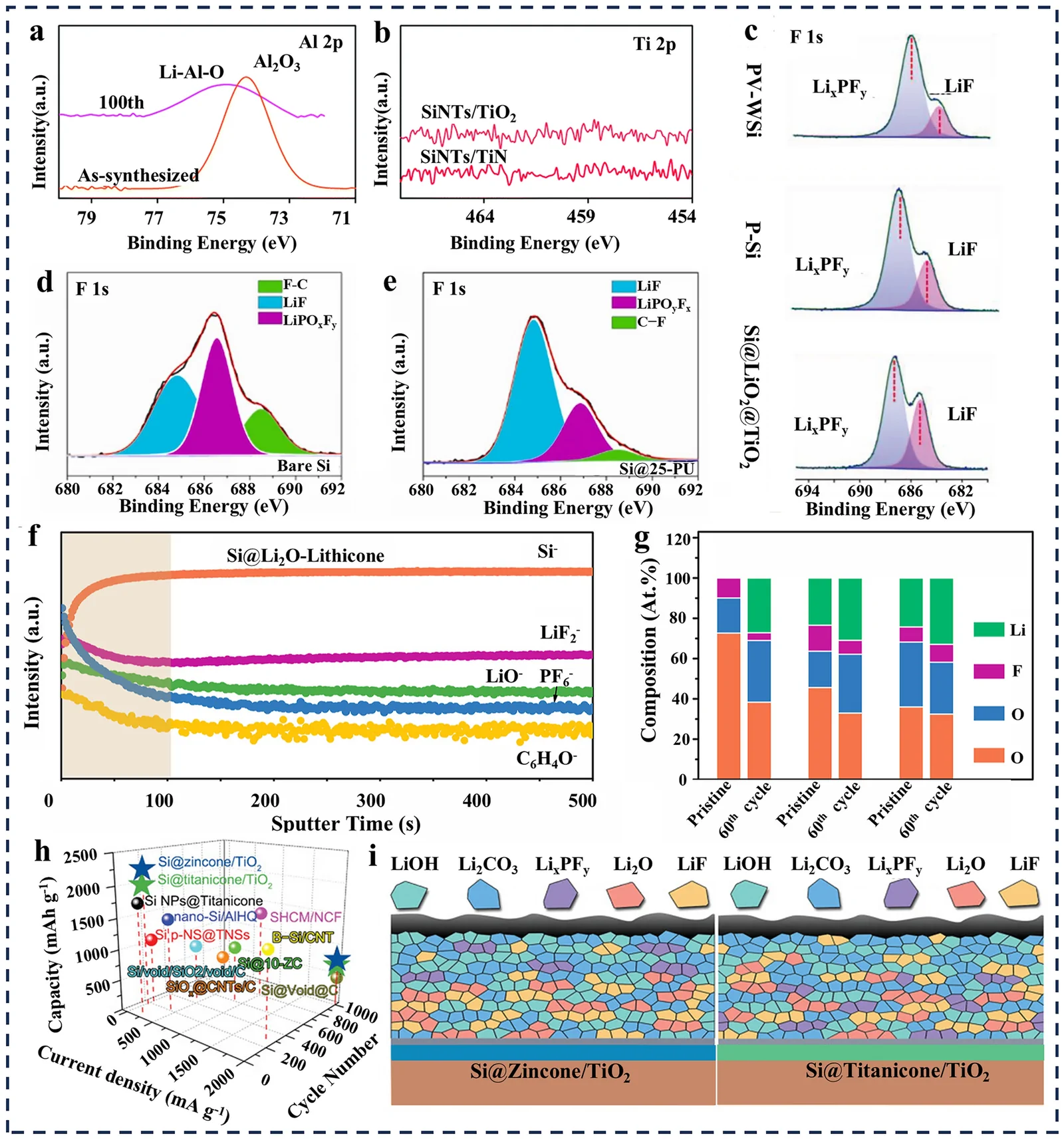
**a** Al 2*p* spectra of SiNTs-coated Al_2_O_3_ electrode after 100 cycles. **b** Ti 2*p* spectra of SiNTs coated with TiO_2_ and TiN. Reproduced with permission [120]. Copyright, 2014 Royal Society of Chemistry. **c** F 1* s* XPS spectra of PV-WSi, P-Si, and Si@Li_2_O@TiO_2_ electrodes after 100 cycles. Reproduced with permission [150]. Copyright 2024, John Wiley and Sons. **d, e** F 1* s* spectra of the electrodes after cycling for the Si and the Si@25-PU electrode. Reproduced with permission [51]. Copyright, 2022 Elsevier. **f** Depth profiles of various secondary ions obtained by sputtering cycles of Si@Li_2_O-lithiconone. **g** Surface composition of the three electrodes: Si, Si@Li_2_O, and Si@Li_2_O–Lithicone. Reproduced with permission [50]. Copyright, 2023 American Chemical Society. **h** Comparison of cycling performance of Si@titanicone/TiO_2_ and Si@zincone/TiO_2_ with other reported Si-based electrodes. **i** Schematic representation of SEI structures formed on Si@zincone/TiO_2_ and Si@titanicone/TiO_2_. Reproduced with permission [143]. Copyright 2021, John Wiley and Sons

Compared with MLD hybrid coatings that can actively participate in the construction of SEI through self-transformation or direct functional groups, inorganic coatings deposited by ALD (such as Al_2_O_3_, TiO_2_) usually play a relatively passive role in regulating the composition of SEI. These coatings are electrochemically inert by Nature Their regulation of the SEI mainly relies on two indirect pathways. One is to act as a physical barrier separating Si from the electrolyte, thereby reducing by-products. The other is to generate new interface phases during lithiation to construct artificial SEI. As reported, the LiTiO_2_ phase derived from TiO_2_ during cycling can enhance the electronic conductivity and promote simple and stable SEI formation, resulting in excellent Coulombic efficiency and capacity retention rate (Fig. 12a, b) [120]. However, this indirect regulation mode that relies on “lithiation products” often has lower efficiency and effectiveness compared to the direct chemical guidance of MLD coatings. To overcome this limitation, researchers have begun to design more sophisticated inorganic composite coating systems. The Li_2_O/TiO_2_ composite coating (Si@Li_2_O@TiO_2_) demonstrates an ingenious functional zoning design [150]. The inner Li_2_O layer serves as a lithium source participating in the LiF formation, while the outer layer TiO_2_ acts as a barrier to decrease side reactions. X-ray photoelectron spectroscopy (XPS) analysis confirms that this strategy successfully induces the enriched LiF formation in SEI, achieving the coordinated regulation of “lithium source compensation-barrier protection” (Fig. 12c).

However, the lithium sources remain confined to the inner layer, and their design principle is partitioned and coordinated. A more comprehensive solution is to construct an overall pre-constructed artificial interface layer rich in active lithium and with uniform organic–inorganic hybridization. Through ALD/MLD, a double-layer structure of Li_2_O and Lithicone was co-deposited on the surface of the Si anode [50]. This design not only achieved a lithium content of up to 24.3% at the interface, but also has the characteristic of organic–inorganic hybridization, which is more conducive to forming a uniform and dense artificial SEI. TOF–SIMS and XPS analyses confirmed that this highly stable lithium-rich interface not only inhibited the continuous decomposition of the electrolyte, but also compensated for the cycle loss by pre-storing active lithium, providing a key guarantee for high ICE and long cycle life (Fig. 12f, g).

A more profound level of interface regulation involves leveraging specific functional groups of polymer molecules to actively “catalyze” the selective induction of the desired SEI component generation pathway. For instance, by introducing a layered charged binder system based on oppositely charged polymers and abundant hydrogen bonds, which constructs a dynamic cross-linked network on the Si surface. The charged functional groups and hydrogen bonds in this system actively participate in regulating SEI formation, promoting the generation of a LiF-rich stable SEI [151]. Similarly, the polyurea coating, containing many hydrogen bonds and polar functional groups, not only ensures strong adhesion to the Si substrate but also plays the role of a “molecular-level interface catalyst”. This coating may actively promote the directional enrichment of LiF through two mechanisms [51]. Firstly, polar groups such as carbonyl (C=O) and amine (N–H) have lone pairs of electrons, which form coordination with Li^+^ through Lewis acid–base interactions, thus concentrating the Li^+^ near the interface and reducing the desolvation energy barrier [152, 153]. DFT calculations show that the binding energy between lithium ions and carbonyl oxygen is comparable to that of typical electrolyte solvents (EC, EMC), enabling the polymer coating to actively participate in the coordination environment of Li^+^ [154]. Secondly, these polar groups will alter the local electronic structure. Electrostatic potential (ESP) calculations indicate that coatings with electron-withdrawing or electron-donating groups will form negative potential regions, attract Li^+^ while repel anions, thereby guiding the decomposition pathways of lithium salts and solvents [155]. Specifically, strong interactions between Li^+^ polar groups reduce the reduction potential of FEC and lithium salts, promoting their preferential decomposition into LiF rather than organic oligomers [154]. XPS analysis (Fig. 12d, e) reveals that the LiF content of Si@PU electrodes after cycling is higher, and it inhibits the formation of undesirable electrolyte reduction products such as LiPO_y_F_x_ [156] and Li_2_CO_3_ [157].

In addition to direct coordination, polar functional groups also promote Li^+^ transport through dynamic coordination-descoordination processes. Li^+^ migrates along the polymer chain by repeatedly binding and dissociating at carbonyl or amine sites, and their activation energy is significantly lower than the activation energy required for diffusion in a thick and disordered SEI layer [158]. Similar functionalities have also been demonstrated in self-assembled molecular layers (SAMLs), where polar terminal groups and fluorinated backbones modulate the electrical double layer and promote LiF-rich SEI formation, underscoring the broader significance of molecular-level chemical design in stabilizing the Si/electrolyte interface [159]. Therefore, polymers with polar functional groups actively guide the formation of stable SEI through multiple mechanisms such as Li^+^ coordination, electronic structure regulation, and reduction of the desolvation energy barrier.

In conclusion, the strategy of regulating SEI through ALD/MLD technology involves: ALD inorganic coatings mainly influence SEI through their inert barrier effect and the products after lithiation. While MLD hybrid coatings directly guide the formation of SEI rich in beneficial components such as LiF through their own electrochemical in situ transformation or specific functional groups. The polar functional groups in the organic polymer coating molecules can actively intervene and optimize the generation path of SEI, achieving the most efficient component regulation.

### Improving Interfacial Charge Transfer Kinetics

The interfacial charge-transfer kinetics of Si anodes, involving the coordinated transport of Li-ions and electrons, is a key determinant of rate capability and fast-charging performance [160]. Slow ion migration and insufficient electron conductivity pathways will lead to severe electrode polarization and capacity degradation. The ALD/MLD technology, by constructing interface layers with specific physical and chemical properties, provides a powerful tool for precisely constructing efficient ion channels and significantly improving the interface charge transport dynamics.

**Fig. 13:**
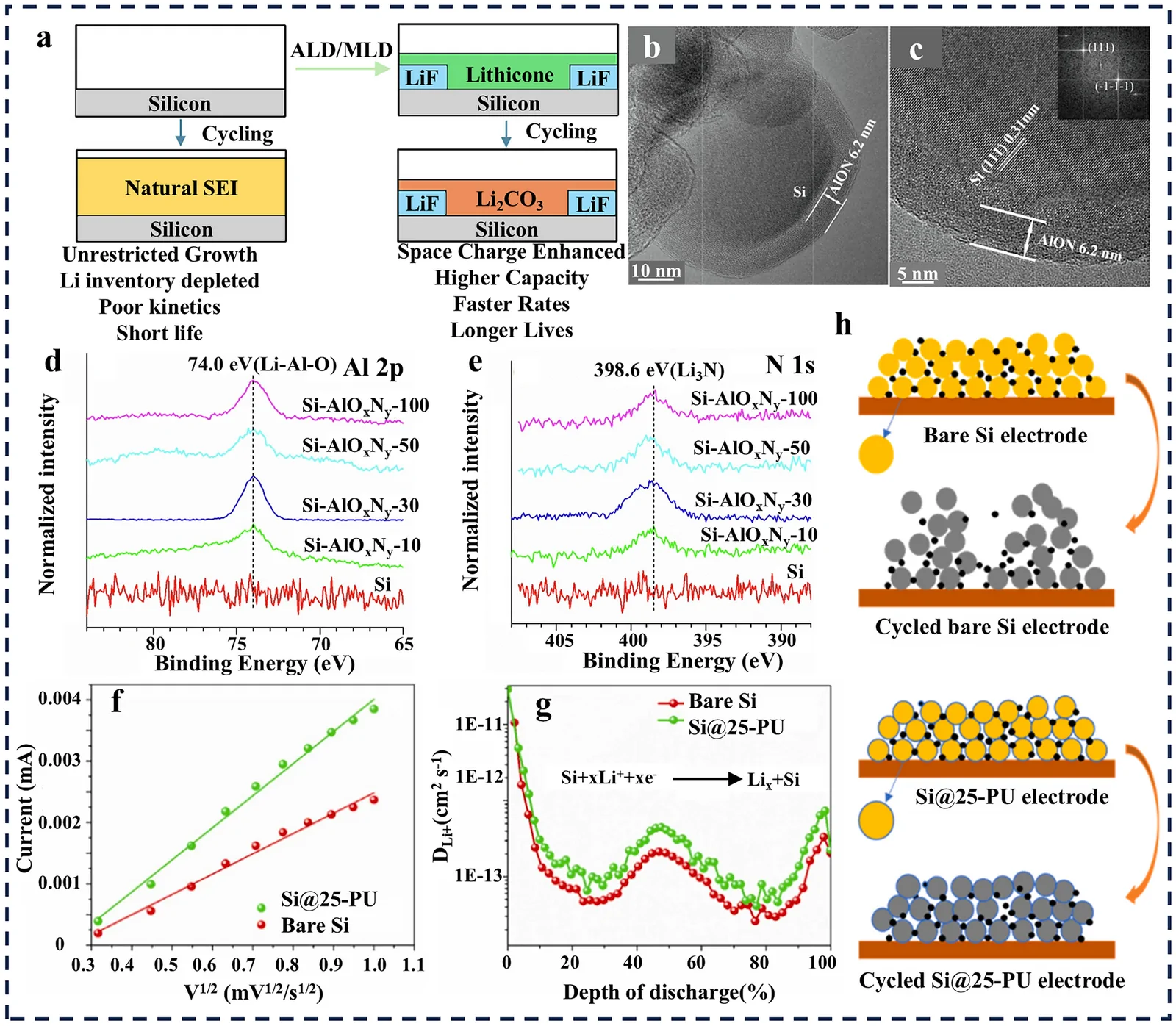
**a** Schematic of the structure of SEI after the cycling of lithicone and LiF coatings. Reproduced with permission [170]. Copyright, 2025 American Chemical Society. **b, c** TEM and HRTEM images of the Si electrode with deposited AlO_x_N_y_ coating. **d, e** XPS spectra of the Si electrodes coated with AlO_x_N_y_ layer after 100 cycles. Reproduced with permission [167]. Copyright, 2022 Elsevier. **f** Linear relationship between the peak current (I_p_) of Si@25-PU and bare Si electrodes and the square root of the scan rate (ν^1/2^). **g** Li^+^ diffusion coefficients of Si@25-PU and bare Si electrodes during lithiation. **h** Schematic showing the structural evolution of bare Si electrode and Si@25-PU electrode before and after cycling. Reproduced with permission [51]. Copyright, 2022 Elsevier

To further break through the electrode kinetics bottleneck, transition metal nitrides with high conductivity and chemical stability have been specifically developed. These materials demonstrate unique advantages in energy storage studies [161–163]. Titanium nitride (TiN) nanoparticles are used as a conductive additive in lithium-ion batteries [164]. Moreover, the combination of TiN and Si shows improved conductivity and enhanced rate performance [165]. Besides TiN coating, nitrogen oxide aluminum (AlO_x_N_y_) has elicited extensive electrical and optical responses [166]. Researchers employed the plasma-enhanced atomic layer deposition (PEALD) technique to fabricate an ultrathin AlO_x_N_y_ coating on Si electrodes (Fig. 13b, c) [167]. It effectively inhibits the electrolyte decomposition and the electrode detachment by physically confining the volume changes of Si particles and chemically regulating the composition of the SEI layer. The XPS analysis after cycling reveals the interfacial chemical interaction between the coating in the lithiation process. The Li–N bond signal at N 1 s peak corresponds to the formation of Li_3_N, while the Li–Al–O bond signal at the Al 2*p* peak indicates that lithium ions react with the coating to form an artificial SEI layer comprising Li_3_N (σ > 1 × 10^–3^ S cm^–1^) with high lithium-ion conductivity and mechanically stable Li–Al–O phases (Fig. 13d, e) [168]. Importantly, the in situ formed Li_3_N not only provides a rapid ion conduction pathway, but also reduces the local charge transfer resistance at the interface by lowering the migration energy barrier of Li^+^ through the SEI. At the same time, the Li–Al–O phase forms a high mechanical strength framework that can maintain the integrity of the electrode structure over long cycles, thereby indirectly protecting the original electronic conductive network. Similarly, studies have reported that the Li_4_Ti_5_O_12_ coating on SiNPs undergoes in situ transformation into the Li_7_Ti_5_O_12_ phase after the first lithiation. This transformed phase exhibits significantly enhanced electronic conductivity while providing rapid diffusion channels for lithium ions, contributing to excellent long-term cycling stability and rate performance [169].

Additionally, the numerous hydrogen bonds and polar functional groups of polyurea coating help it to achieve strong adhesion to the Si surface and facilitate the kinetics of Li^+^ transfer, making it an appropriate artificial polymer interface. The multi-scan CV and GITT tests also verify the adsorption and transfer of lithium ions through dynamic coordination, which is more conducive to the electrochemical reaction kinetics of the alloying process (Fig. 13f–h) [51]. From a microscopic perspective, the dynamic coordination interaction between Li^+^ and polar groups reduces the resistance of charge transfer at the interface by providing low-energy barrier ion jumping sites. At the same time, these polar regions can regulate the local distribution of Li^+^ and the related spatial charge regions, thereby influencing the potential distribution at the interface, making it possible for electrons to maintain transmission through the tunneling mechanism within the ultrathin polymer layer, and indirectly protecting the continuity of the electron pathway. Although the MLD polymer coatings perform well in optimizing ion transport, their electronic conductivity is usually limited. To simultaneously enhance the ion and electron conductivity at the interface, Pope et al. combined LiF with Lithicone to construct a hybrid artificial SEI, thereby creating a multifunctional composite interface and achieving synergistic enhancement of ion and electron transport [170]. It is worth noting that during cycling, the Lithicone coating transforms into Li_2_CO_3_, which together with LiF forms a mosaic-like structure rich in the favorable LiF/Li_2_CO_3_ interface. This interface structure has been proven to significantly enhance the space-charge effect on the Si surface. Specifically, Li^+^ is driven by the chemical potential difference from LiF into the interstitial sites of Li_2_CO_3_, increasing the concentration of mobile Li^+^. Concurrently, the resulting localized charge imbalance creates a capacitor-like effect that suppresses electron leakage associated with SEI growth. This synergistically improves interfacial ion transport efficiency and stabilizes the SEI (Fig. 13a). From an electronic perspective, the LiF/Li_2_CO_3_ composite interface introduces a high electron barrier, which hinders the electron tunneling from the Si surface into the SEI, thereby inhibiting the decomposition of the electrolyte. This electron confinement effect indirectly protects the electronic conductive network by minimizing the formation of insulating by-products, while enhancing ion transport through the space charge region and achieving effective decoupling of ion and electron transport paths, thereby maximizing the overall transmission efficiency.

**Fig. 14:**
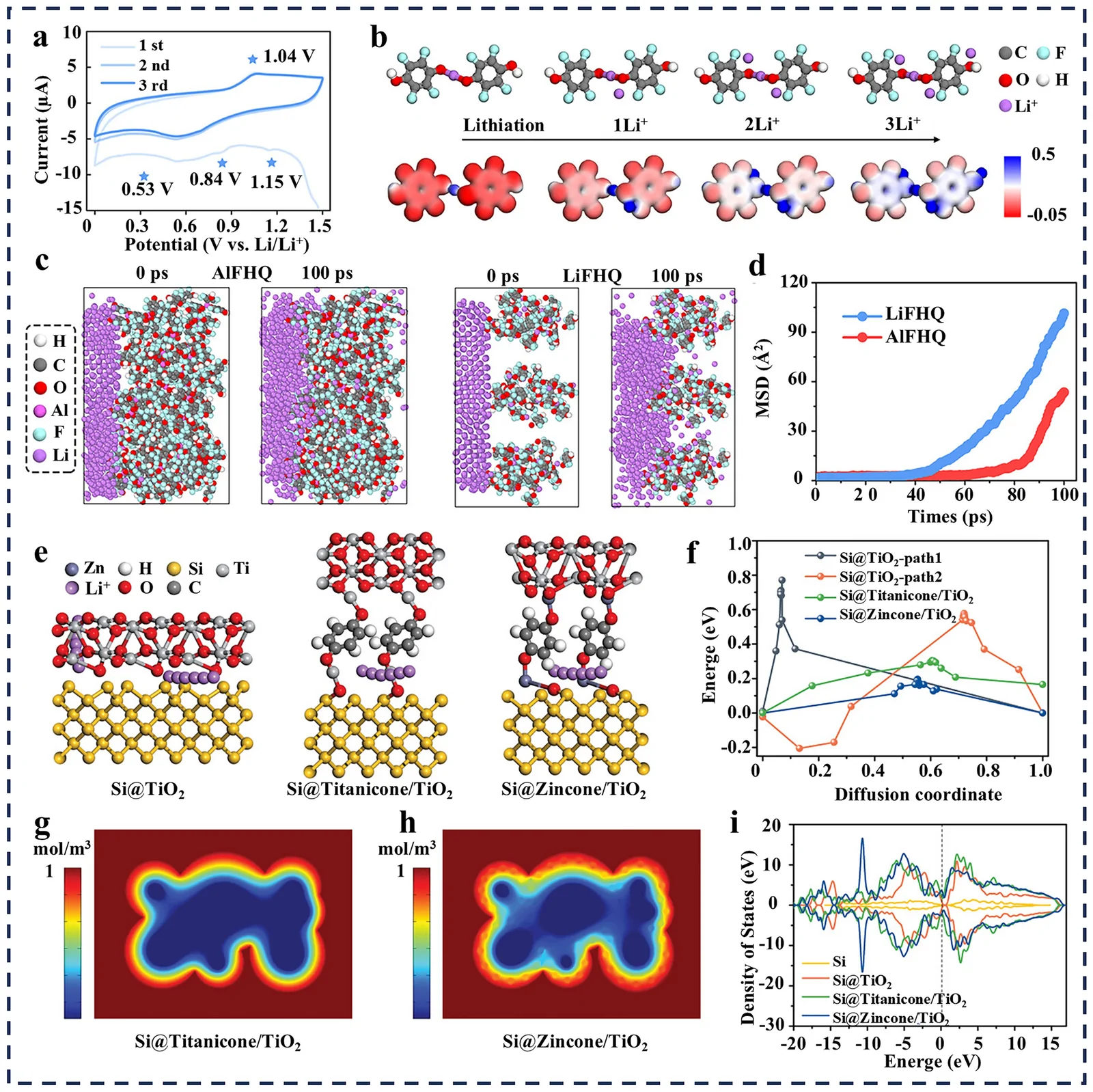
**a** CV curve of LiFHQ coating. **b** Optimized Li^+^ adsorption configuration and corresponding ESP diagram of LiFHQ system. **c** Snapshots of the simulation trajectory at 0 and 100 ps. **d** MSD of Li^+^ diffusion. Reproduced with permission [171]. Copyright 2025, John Wiley and Sons. **e** Li^+^ migration path and **f** corresponding diffusion energy barrier profiles. Concentration distribution of Li^+^ ions in **g** Si@titanicone/TiO_2_ and **h** Si@zincone/TiO_2_. **i** Total density of states of Si, Si@TiO_2_, Si@titanicone/TiO_2_, and Si@zincone/TiO_2_ electrodes. Reproduced with permission [143]. Copyright 2021, John Wiley and Sons

However, excellent interface dynamics require rapid migration of ions within the solid phase. More importantly, they also demand a reduction of the desolvation energy barrier when Li^+^ migrates from the electrolyte into the solid-phase interface. Therefore, some researchers have used MLD to deposit LiFHQ on the surface of Si-carbon composite (CB@Si), and from the perspective of Li^+^ desolvation, they have revealed the enhancement mechanism of the coating on interface charge transport [171]. As shown in the CV curve of the LiFHQ coating deposited on the copper foil in Fig. 14a, during the first reduction process, there are three irreversible peaks at 1.15, 0.84, and 0.53 V, corresponding to the multi-step lithiation process of LiFHQ. After the initial activation, the coating transforms into a stable mixed interface layer composed of a SEI rich in LiF and a porous LiFHQ framework. It is noteworthy that this activated framework is not static but shows reversible structural evolution in subsequent cycles, which directly helps to improve the transport of Li^+^. In the deep lithiation state, the porous LiFHQ absorbs stress through pore compression; upon Si delithiation and contraction, the pores undergo elastic recovery, ensuring the structural integrity of the coating during long-term cycling.

The uniform, conformal, and molecularly precise LiFHQ layer achieved by MLD is central to this mechanism. The self-limiting, layer-by-layer growth ensures that fluorinated and other functional groups are anchored at a precisely controlled density and spatial arrangement across the entire complex Si-carbon surface. This level of uniformity and structural control is exceedingly difficult to achieve through alternative methods like solution-phase functionalization or the use of fluorinated electrolyte additives, which often lead to heterogeneous, poorly adhered, or kinetically unstable interfaces [172, 173]. For instance, conventional additives like fluoroethylene carbonate (FEC) face high reduction barriers, resulting in incomplete decomposition and the formation of a disordered LiF distribution during initial cycles [174]. Moreover, these additive-based approaches are often accompanied by side reactions that hinder the optimal performance of Si anodes [175].

The electrostatic potential calculation (Fig. 14b) indicates that the F and O atoms in the LiFHQ molecular layer act as powerful nucleophilic sites, which can directly anchor and activate lithium ions in the electrolyte. It is believed that this can significantly reduce the key Li^+^ desolvation energy barrier. At the same time, the molecular dynamics simulation (Fig. 14c, d) shows that Li^+^ can rapidly transport preferentially in the interconnected nanopores of LiFHQ. The well-defined, continuous nanoporosity revealed by the simulation is a direct consequence of the ordered molecular packing enabled by the MLD process, a structural feature not guaranteed by conventional coating techniques. This enables the coating to synergistically optimize the “desolvation-solid phase migration” continuous process, thereby fundamentally improving the interface reaction kinetics.

Another study employed systematic DFT calculations and finite element simulations to analyze the underlying reasons for the synergistic enhancement of kinetics by ALD/MLD [143]. The comparative study of Si@TiO_2_, Si@titanicone/TiO_2_, and Si@zincone/TiO_2_ demonstrated that the internal porous zincone layer provided an extremely low migration energy barrier for Li^+^, which was significantly better than the dense titanicone and the pure TiO_2_ coating (Fig. 14e, f). Furthermore, the density of states analysis showed that the hybrid coating enhanced the electron density near the Fermi level and strengthened the electron conductivity (Fig. 14i). The finite element simulation further confirmed that this structure could effectively reduce the Li^+^ depletion zone and achieve a more uniform ion concentration distribution (Fig. 14g, h). The key point is that the gradient structure forms an intrinsic electric field at the zincone/TiO_2_ heterojunction. This electric field can drive Li^+^ through the interface, while creating an electron barrier to inhibit electron passage, thereby generating a rectification effect of ion/electron selective transport, enhancing the ion transfer kinetics and reducing the side reactions caused by electron leakage. These results comprehensively revealed the synergistic mechanism of the flexible-rigid heterogeneous structure for efficient charge transport from three dimensions: ion migration energy barrier, electron conductivity, and macroscopic ion flux.

In summary, the enhanced charge transfer kinetics achieved by ALD/MLD originate from a multiscale interplay between ion transport regulation and electronic network stabilization. At the molecular level, functional groups such as C−F, C=O, and −NH− reconfigure the coordination environment of Li^+^, lowering the desolvation energy barrier, while ordered molecular packing creates low-tortuosity nanopores that facilitate ion migration. At the nanoscale, the in situ formed phases each serve a distinct function: Li_3_N acts as a fast ionic conductor, constructing efficient ion transport pathways; Li−Al−O forms a mechanically robust framework that maintains electrode structural integrity during volume cycling; and the LiF/Li_2_CO_3_ heterointerface introduces a high electron barrier that suppresses electron leakage, reducing the accumulation of insulating by-products and thereby preserving the electronic percolation network. Crucially, these mechanisms are interrelated, and the optimization of molecular and nanoscale ion transport requires continuous electron supply at the reaction front to avoid polarization, which is achieved through the maintenance and enhancement of the aforementioned electron network. The ALD/MLD technology, with its atomic/molecular scale precision, realizes this multi-scale collaborative design, fundamentally addressing the multi-faceted kinetic limitations of the Si anodes.

## Summary and Perspectives

### Summary

This review has systematically deconstructed the interfacial failure mechanisms of Si anodes, which stem from substantial volume changes and manifest as three coupled core challenges: mechanical failure and structural degradation, complex/unstable SEI, and insufficient interfacial charge transfer. We articulate how ALD/MLD serves as a precision engineering platform to address these issues. By enabling atomic-scale control over thickness, conformality, and composition, ALD/MLD transcends simple passivation, allowing for the rational design of multifunctional interfaces. Specifically, it effectively suppresses mechanical failure, directs SEI evolution, and enhances charge transfer kinetics through the construction of diverse structures including rigid inorganic coatings, flexible hybrid “metalcones”, functional polymers, and gradient composites. This capability directly tackles the coupled chemo-mechanical challenges at their root.

It is worth noting that most of the reported applications of ALD/MLD in the interface engineering of Si anodes are carried out on the surface of pre-fabricated Si electrodes (Table 2). Although this strategy effectively avoids inherent problems such as particle agglomeration during powder coating, it also means that exploration of the powder-level ALD/MLD coating process remains relatively limited. At the same time, existing research is highly concentrated on nano-sized Si, mainly due to the smaller volume expansion stress and lower interface failure risk at the nanoscale. In contrast, micro-sized Si, although having advantages such as high tap density and low cost for industrialization, undergoes more intense volume changes, which impose higher requirements on the mechanical properties of the interface protective layer. Relevant research remains insufficient. Moreover, existing literature pays little attention to the failure mechanism of the ALD/MLD coating itself. Most studies focus on the improvement of electrochemical performance, while systematic research on potential failure modes, such as fatigue cracking of the coating during long-term cycling and chemical instability under extreme fast-charging conditions, is lacking. Therefore, to further accelerate the practical application and commercialization of ALD/MLD in the interface engineering of Si anodes, it is particularly necessary to systematically review the current challenges and future development directions in this field.

### Perspective

1. Mechanism of Microscopic Electronic Interaction

As discussed above, significant progress has been made in the regulation of the Si anode interface by MLD. However, the study of the microscopic electronic interactions between lithium ions and the surface molecular layer during lithium intercalation remains in its infancy. In fact, the orbital hybridization and charge transfer that occur between lithium ions and the interface functional groups directly reconstruct the orbital energy levels (HOMO/LUMO) of the interface molecular layer, thereby affecting the reductive decomposition behavior of the electrolyte on the surface [176]. Recent related studies have provided a new electronic structure perspective for understanding the mechanism of the molecular interface layer [177, 178].

In view of the aforementioned points, the future design of MLD-deposited molecular layer interfaces should go beyond traditional thinking and focus on orbital energy level regulation. For example, DFT calculations can be used to screen and construct functional groups and molecular configurations with ideal electronic structures. Meanwhile, in situ ultraviolet photoelectron spectroscopy (UPS) and in situ XPS can be combined to reveal the dynamic evolution of the chemical state and energy levels of the MLD molecular layer during cycling.

(2) Failure Mechanisms of ALD/MLD-Derived Interfaces

Currently, most studies focus on the improvement of the electrochemical performance of Si anodes by ALD/MLD coatings, while paying little attention to the physicochemical properties and failure behaviors of the coatings themselves. Systematic characterization of key parameters, such as mechanical properties, ionic conductivity, electronic insulating properties, and chemical stability, remains insufficient. Due to the lack of understanding of the intrinsic properties of these coatings, the mechanical fatigue behavior during long-term cycling, as well as whether the local overpotential and Joule heating induced by high current densities under extreme fast-charging conditions accelerate chemical side reactions at the coating/electrolyte interface, have not been systematically investigated. In addition, the impact of different coatings on organic electrolyte consumption has rarely been quantitatively evaluated.

It is believed that future research should establish quantitative characterization methods for the intrinsic properties of these coatings, including elastic modulus, roughness, ionic conductivity, and chemical stability. In situ mechanical testing and spectroscopic characterization should be employed to reveal the failure thresholds and evolution mechanisms of coatings under long-term cycling and extreme fast-charging conditions. Furthermore, techniques such as in situ gas chromatography or nuclear magnetic resonance should be introduced to quantitatively assess the impact of different coatings on electrolyte consumption.

(3) Application of High-Entropy Oxides in Interface Engineering

The emerging high-entropy oxides (HEOs) offer a new perspective for ALD interface engineering. Unlike conventional single-component inorganic coatings, HEOs exhibit multiple unique advantages that address the specific requirements of Si anodes through the synergistic interplay of the high-entropy effect, lattice distortion effect, sluggish diffusion effect [179, 180]. For instance, the synergistic design of multi-principal elements enables simultaneous suppression of electrolyte decomposition and rapid ion transport, circumventing the trade-off between protection and ion transport inherent to conventional coatings. Recent studies have confirmed that HEO coatings can effectively inhibit lattice crack formation in cathode materials, significantly improving cycling stability [181, 182].

In light of these advances, future research should focus on the following directions: drawing inspiration from the composition design strategies of traditional coating approaches, combined with first-principles calculations and high-throughput screening, to identify HEO compositions with high lithium-ion conductivity, excellent toughness, and electrochemical stability; simultaneously, developing ALD processes suitable for high-entropy oxide films to address key challenges such as matching of reaction windows and compositional uniformity. Techniques such as PEALD and ALD combined with rapid thermal annealing (ALD-RTA) can be leveraged to achieve uniform and conformal deposition of high-entropy coatings.

(4) Tailored ALD/MLD Strategies for Micro-Sized Si

It is well known that micro-sized Si particles are favored for commercial applications due to their high tap density and low cost. However, micro-sized Si faces more severe volume expansion during lithiation, which places higher demands on the mechanical properties of the interface protective layer. In recent years, the “flexible skin” design strategy (double conductive polymer layers, double carbon layer matrix, cross-linked polymer sealing layer) has provided important insights for addressing the interface stability issue of micro-sized: the stability of the micro-sized Si interface requires a stable “dynamic interface” [183–185].

When using ALD/MLD technology to construct this dynamic interface, the following directions should be explored: by regulating the molecular chain length and cross-linking density of the MLD precursor molecules, an elastic interface layer with excellent elastic modulus and reversible deformation capability can be constructed, ensuring the integrity of the electrode. Furthermore, a multi-layer gradient structure can be designed, with the inner layer being a flexible polymer layer to dissipate stress and the outer layer being a dense inorganic layer to seal off the electrolyte. Additionally, for the geometric characteristics of micro-sized Si particles, ALD/MLD reaction chambers suitable for powder particles, such as rotating beds or fluidized beds, should be developed to ensure uniform and complete coating coverage on the surface of micro-sized Si and to achieve batch processing.

(5) Large-Scale Application of ALD/MLD Processes

In view of Si anodes, the industrial application of ALD/MLD technology faces low deposition efficiency and difficulty in balancing throughput and cost. To break through this bottleneck, some effort must be made in both process selection and system design. The ALD/MLD technology can be applied to both particle-level coating and electrode-level coating, each with its own advantages and disadvantages. Particle-level coating enables conformal encapsulation of individual particles with excellent coating uniformity, but requires powder reactors such as fluidized beds and rotating beds, which have limited processing throughput [186]. Electrode-level coating is performed after electrode fabrication, offering a simpler process and better compatibility with existing battery production lines, making uniform coating of all particle surfaces challenging.

To address these limitations, for particle-level coating, the principle of spatial ALD can be adopted, converting the traditional time-sequential mode into a spatial separation mode that allows the powder to move continuously between different reaction zones, significantly increasing the deposition rate, and can be combined with fluidized bed or rotating bed reactors to achieve batch processing. For electrode-level coating, roll-to-roll ALD represents a promising technological route. By continuously feeding the current collector through the reaction chamber, the precursor adsorption, purging, and reaction steps are completed sequentially in multiple chambers in series. This technology has been demonstrated in GW-level mass production in the fields of photovoltaics, and can be extended to the large-scale coating of Si anodes.

(6) Application of ALD/MLD to All-Solid-State Batteries

The rise of all-solid-state batteries (SSBs) has brought new challenges and unique opportunities to ALD/MLD. The failure mechanism of Si anodes in liquid systems has been systematically studied, but in solid-state systems, fundamental issues such as insufficient solid–solid interface contact and differences in interface compatibility between different solid-state electrolytes and Si remain to be elucidated. Therefore, to fully leverage the huge potential of ALD/MLD, future research should focus on the following directions: First, in combination with advanced in situ characterization techniques, the intrinsic mechanisms of interface evolution in solid-state batteries should be further revealed.

On this basis, ALD/MLD technology can provide customized interface engineering strategies based on the characteristics of different solid-state electrolyte systems. For sulfide electrolytes, which suffer from poor chemical stability and are prone to electrochemical reduction decomposition at low potentials, ultra-thin Li_3_N or Li_3_PO_4_ fast ion conductor layers can be deposited by ALD, taking advantage of their high ionic conductivity and good chemical compatibility with sulfide electrolytes to construct stable ion transport interfaces. For halide electrolytes, ALD deposition of LiF or MLD deposition of fluorine-containing organic–inorganic hybrid layers can be used to suppress side reactions between halide electrolytes and Si through fluorinated interfaces.
